# Pen-2 regulates glial homeostasis by coordinating self-renewal and transdifferentiation programs in oligodendrocyte precursor cells

**DOI:** 10.1016/j.stemcr.2025.102612

**Published:** 2025-08-28

**Authors:** Huiru Bi, Jinxing Hou, Wenkai Shao, Chenyi Ge, Yang Liu, Runmin Wang, Guiquan Chen, Yun Xu, Zhiye Wang

**Affiliations:** 1 Department of Neurology, Nanjing Drum Tower Hospital, Affiliated Hospital of Nanjing University Medical School, Medical School, Nanjing University, Nanjing 210008, China; 2 Nanjing Normal University of Special Education, Nanjing 210038, China; 3 Department of Laboratory Medicine, Nanjing Drum Tower Hospital, Affiliated Hospital of Medical School, Nanjing University, Nanjing 210008, China; 4 Model Animal Research Center, Jiangsu Key Laboratory of Molecular Medicine, Medical School, Nanjing University, Nanjing 210061, China; 5 Suqian Scientific Research Institute of Nanjing University Medical School, Nanjing University, Nanjing 210008, China; 6 Department of Neurology, Meishan Hospital of Nanjing, Nanjing 210039, China

**Keywords:** Pen-2, ASCL1, STAT3, OPC self-renewal, γ-Secretase

## Abstract

Presenilin enhancer 2 (Pen-2) is implicated in neurological diseases characterized by glial dysregulation. To investigate whether oligodendrocytic γ-secretase activity is important for the maintenance of glial populations, we analyzed two conditional knockout (cKO) mouse models lacking Pen-2 or nicastrin. Both models exhibited similar expansions of oligodendrocyte precursor cells (OPCs) and astrocytes in the CNS. To test whether STAT3 mediates Pen-2-dependent glial homeostasis, we inactivated *Stat3 in Pen-2* cKO mice. Intriguingly, STAT3 deficiency did not attenuate OPC expansion but normalized astrocyte numbers. We further demonstrated that Pen-2 represses *Ascl1* expression via HES1 and that *Ascl1* knockdown rescues the aberrant self-renewal capacity of Pen-2-deficient OPCs. Collectively, these results uncover a dual regulatory mechanism by which Pen-2 maintains glial homeostasis by (1) restraining OPC self-renewal through the HES1-ASCL1 axis and (2) suppressing OPC-to-astrocyte transdifferentiation in a STAT3-dependent manner. Our findings provide novel insights into glial abnormalities in PEN-2-linked neurological diseases.

## Highlights

• OL-lineage-specific inactivation of Pen-2 disrupts glial homeostasis in the CNS
• Pen-2 transcriptionally regulates *Ascl1* expression via HES1
• Knockdown of *Ascl1* restores aberrant self-renewal in Pen-2-deficient OPCs
• STAT3 mediates Pen-2-dependent OPC-to-astrocyte transdifferentiation but not OPC self-renewal

## Teaser

In this article, Bi and colleagues investigate the role of oligodendrocytic Pen-2 in maintaining glial homeostasis, uncovering a dual regulatory mechanism. They demonstrate that Pen-2 not only inhibits oligodendrocyte precursor cell (OPC) self-renewal via the HES1-ASCL1 axis but also suppresses OPC-to-astrocyte transdifferentiation in a STAT3-dependent manner.

## Introduction

Astrocytes and oligodendrocytes (OLs) are two major types of glial cells in the mammalian central nervous system (CNS), where they perform essential physiological functions (Lee et al., 2023; Liu et al., 2023). It is known that glial cells constitute approximately 50% of all cells in the adult human brain, with astrocytes and OLs present in roughly equal numbers (von Bartheld et al., 2016). While glial populations are relatively stable under healthy conditions, they are often significantly altered in neurological disorders. Notably, increasing evidence shows a marked rise in astrocyte numbers in neurodegenerative disorders such as Alzheimer disease (AD), Parkinson disease (PD), Huntington disease (HD), and multiple sclerosis (MS) (Lee et al., 2023; Sadick et al., 2022). However, the mechanisms driving these changes remain poorly understood.

During brain development, intermediate neural progenitor cells (NPCs) give rise to astrocytes, while oligodendrocyte (OL) precursor cells (OPCs) either self-renew or differentiate into mature OLs (Rowitch and Kriegstein, 2010). Therefore, the regulation of glial progenitor proliferation and differentiation is critical for maintaining glial population homeostasis, and impairments in these processes may result in disrupted numbers of OPCs, Ols, and astrocytes. Several transcription factors (TFs), including SCL, SOX9, NFIA, STAT3, and TCF4, have been shown to play pivotal roles in the differentiation of astrocytes from NPCs (Deneen et al., 2006; Fan et al., 2005). Similarly, SOX10 and MYRF are critical for OL differentiation, and their downregulation leads to a significant reduction in mature OLs (Stolt et al., 2002; Teng et al., 2024; Wang et al., 2021). In addition to transcriptional control, cell death mechanisms may also substantially influence glial populations. Given that OPCs have the potential to differentiate into OLs, astrocytes, and neurons (Guo et al., 2010; Zhu et al., 2012), their multipotency positions them as key regulators of glial balance in the CNS. Notably, recent studies have demonstrated that OL-derived molecules, including OLIG2, HDAC3, and RBPj, are critical for the regulation of both astrocyte and OL populations (Guo et al., 2023; Zhang et al., 2016; Zhu et al., 2012).

Presenilin enhancer 2 (PSENEN, abbreviated as Pen-2 in this study) is an essential subunit of γ-secretase, a protease that cleaves type I membrane proteins such as Notch receptors and amyloid precursor protein (APP) (Steiner et al., 2002). Accumulating evidence has linked PEN-2 dysfunction to neurological diseases characterized by abnormal glial populations in the brain (Albani et al., 2007; Gana et al., 2012). Emerging evidence suggests that Pen-2 has both γ-secretase-dependent (Steiner et al., 2002) and γ-secretase-independent roles (Ma et al., 2022). Deletion of Pen-2 in OL lineage cells has been shown to cause upregulation of STAT3 and significant alterations in astrocyte numbers in the cortex of immature mice (Hou et al., 2021), but whether this phenotype depends on Pen-2’s γ-secretase activity is unknown. Interestingly, nicastrin (NCSTN), another γ-secretase subunit that serves as a gatekeeper for substrate binding (Shah et al., 2005), has no reported γ-secretase-independent functions. Thus, a direct comparison between OL-lineage-specific *Pen-2* cKO and *Ncstn* cKO mice may help determine whether γ-secretase activity is critical for maintaining glial homeostasis. In addition, STAT3 has been implicated in OPC proliferation and differentiation (Hackett et al., 2016; Steelman et al., 2016). To investigate whether STAT3 mediates the expansions of OPC and astrocyte populations in *Pen-2* cKO mice, we performed *in vivo* and *in vitro* studies. Our findings suggest that Pen-2 regulates glial homeostasis by coordinating the ASCL1-dependent self-renewal and STAT3-mediated transdifferentiation pathways.

## Results

### Oligodendrocytic Pen-2 regulates OPC and astrocyte populations through a γ-secretase-dependent pathway

**Figure 1:**
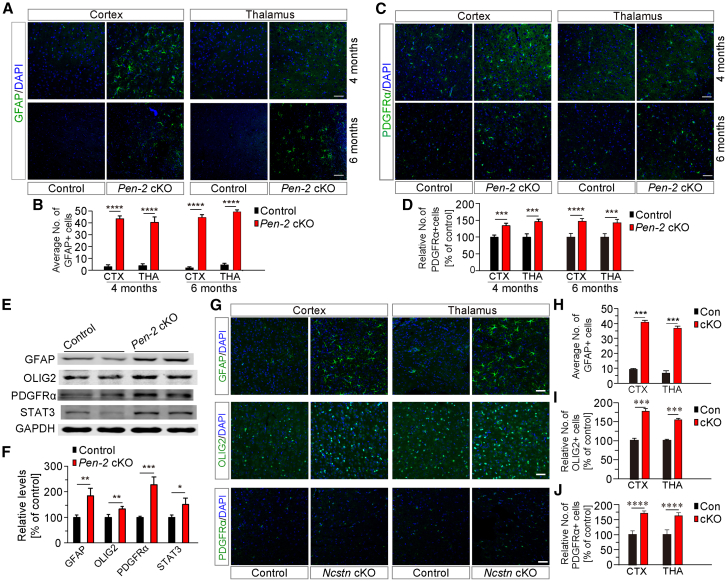
Increased numbers of astrocytes and OPCs in OL-lineage-specific *Pen-2* and *Ncstn* cKO mice (A) Representative fluorescence IHC images for GFAP in control and OL-lineage-specific *Pen-2* cKO mice. Brain sections from mice at 4 and 6 months were used. Scale bars, 50 μm. (B) Quantification of the average number of GFAP+ cells in the cortex and thalamus. Significant differences were observed between control and *Pen-2* cKO mice (*n* = 4 mice per group; ^∗∗∗∗^*p* < 0.0001). (C) Representative fluorescence IHC images for PDGFRα in OL-lineage-specific *Pen-2* cKO mice at 4 and 6 months. Scale bars, 50 μm. (D) Quantification of the relative number of PDGFRα+ cells in the cortex and thalamus. Significant differences were observed between control and *Pen-2* cKO mice (*n* = 4 mice per group; ^∗∗∗^*p* < 0.001). (E and F) Western blotting analysis of GFAP, OLIG2, PDGFRα, and STAT3. Cortical samples from control and *Pen-2* cKO mice at 4 months were used (E). Relative protein levels of GFAP, OLIG2, PDGFRα, or STAT3 were significantly increased in *Pen-2* cKO mice compared with controls (*n* = 4 mice per group; ^∗^*p* < 0.05; ^∗∗^*p* < 0.01; ^∗∗∗^*p* < 0.001) (F). (G) Representative fluorescence IHC images for GFAP, OLIG2, and PDGFRα in control and OL-lineage-specific *Ncstn* cKO mice. Scale bar, 50 μm. (H) Quantification of the average number of GFAP+ cells in the cortex and thalamus. Significant differences were observed between control and *Ncstn* cKO mice (*n* = 4 mice per group; ^∗∗∗^*p* < 0.001). (I) Quantification of the relative number of OLIG2+ cells. Significant differences were observed between control and *Ncstn* cKO mice (*n* = 4 mice per group; ∗∗∗, *p* < 0.001). (J) Quantification of the relative number of PDGFRα+ cells. Significant differences were observed between control and *Ncstn* cKO mice (*n* = 4 mice per group; ∗∗∗∗, *p* < 0.0001).

We have recently reported that conditional inactivation of oligodendrocytic Pen-2 increases the number of glial fibrillary acidic protein (GFAP)-positive (GFAP+) astrocytes in the mouse cortex at early developmental stages such as postnatal day 11 (P11) or P14 (Hou et al., 2021). Since a previous study showed that loss of presenilin1 (PS1) only results in a transient increase in GFAP+ cells in the mouse brain (Sardi et al., 2006), given both Pen-2 and PS1 are essential subunits of γ-secretase (Steiner et al., 2002), we hypothesized that Pen-2 may regulate astrogliogenesis in an age-dependent manner. To test it, we conducted morphological and biochemical analyses using OL-lineage-specific *Pen-2* cKO mice aged at 4 and 6 months. First, fluorescence immunohistochemistry (IHC) revealed qualitatively enhanced immunoreactivity of GFAP in the cortex and the thalamus of *Pen-2* cKO mice (Figure 1A). The average number of GFAP+ cells was significantly increased in these two regions in *Pen-2* cKO brains compared with littermate controls at each age (Figure 1B), indicating enhanced astrogliogenesis. Second, to assess changes in OPCs, PDGFRα was used as a marker (Wang et al., 2021) for fluorescence IHC (Figure 1C). Cell counting results demonstrated that the average number of PDGFRα+ cells was significantly increased in the cortex or the thalamus of *Pen-2* cKO mice compared with age-matched controls at each age (Figure 1D), suggesting expanded OPC population in the CNS of *Pen-2* cKO mice. Third, western blotting was performed to examine markers for astrocytes and OPCs (Figure 1E). We observed that cortical protein levels of GFAP, OLIG2, PDGFRα, and STAT3 were significantly elevated in *Pen-2* cKO mice compared with littermate controls at 4 months (Figure 1F). Overall, these biochemical and histological results were consistent, indicating significantly disrupted glial populations in the cortex of mature *Pen-2* cKO mice.

Given glial homeostasis involves complex cross-talk with microglia, pericytes, and endothelial cells, we further performed IHC on *Pen-2* cKO brain sections using markers for these cell types (IBA1 for microglia, PDGFRβ for pericytes, and CD31 for endothelial cells). However, cell quantification revealed no significant differences in the numbers of IBA1+, PDGFRβ+, and CD31^+^ cells between control and *Pen-2* cKO mice (Figures S1A–S1D), suggesting that OL-lineage-specific deletion of Pen-2 does not affect the populations of microglia, pericytes, and endothelial cells in the cortex.

Since Pen-2 possesses both γ-secretase-dependent (Steiner et al., 2002) and γ-secretase-independent functions (Ma et al., 2022), we investigated whether glial homeostasis requires γ-secretase activity. Given that NCSTN is a substrate-binding subunit of γ-secretase (Shah et al., 2005) and has not been reported to exhibit γ-secretase-independent functions, we generated OL-lineage-specific *Ncstn* cKO mice (Figure S1E) using floxed *Ncstn* mice reported previously (Tabuchi et al., 2009). To assess the KO efficiency for NCSTN, western blotting was performed using cortical lysates prepared from *Ncstn* cKO mice at P30. A significant reduction in NCSTN protein levels was observed in *Ncstn* cKO mice compared with littermate controls (Figure S1F), indicating efficient inactivation of NCSTN. Nissl staining revealed comparable brain morphology between control and *Ncstn* cKO mice (Figure S1G). IHC for NeuN was conducted, and no significant reduction in the average number of NeuN+ cells was observed in the cortex of *Ncstn* cKO mice compared with littermate controls (Figures S1H and S1I). Western blotting further confirmed unchanged cortical levels of NeuN in *Ncstn* cKO mice compared with littermate controls (Figures S1J and S1K).

Next, we performed IHC for GFAP, glutamine synthetase (GS), OLIG2, and PDGFRα. First, qualitatively increased immunoreactivity of GFAP was observed in the cortex and thalamus of *Ncstn* cKO mice compared with littermate controls (Figure 1G). Quantification results revealed a significantly increased number of GFAP+ cells in the cortex and thalamus of *Ncstn* cKO mice compared with littermate controls (Figure 1H). Second, IHC analysis showed significantly increased average number of GS + cells in the cortex of *Ncstn* cKO mice compared with littermate controls at P30 (Figures S1L and S1M), again suggesting enlarged astrocyte population. In addition, western blotting confirmed significantly elevated levels of GFAP and STAT3 in *Ncstn* cKO mice compared with littermate controls (Figures S1J and S1K). Third, IHC data further demonstrated that the average number of OLIG2+ or PDGFRα+ cells was significantly increased in the cortex and thalamus of *Ncstn* cKO mice compared with controls (Figures 1G, 1I, and 1J), suggesting an expanded OPC population in *Ncstn* cKO mice. Overall, the phenotypes in astrocytes and OPCs were identical between *Pen-2* cKO and *Ncstn* cKO mice. Given that NCSTN has not been reported to exhibit γ-secretase-independent functions, these results suggest that oligodendrocytic γ-secretase activity is important for maintaining glial homeostasis in the cortex.

### Stat3 mediates the expansion of astrocytes, but not OPCs, in *Pen-2* cKO mice

**Figure 2:**
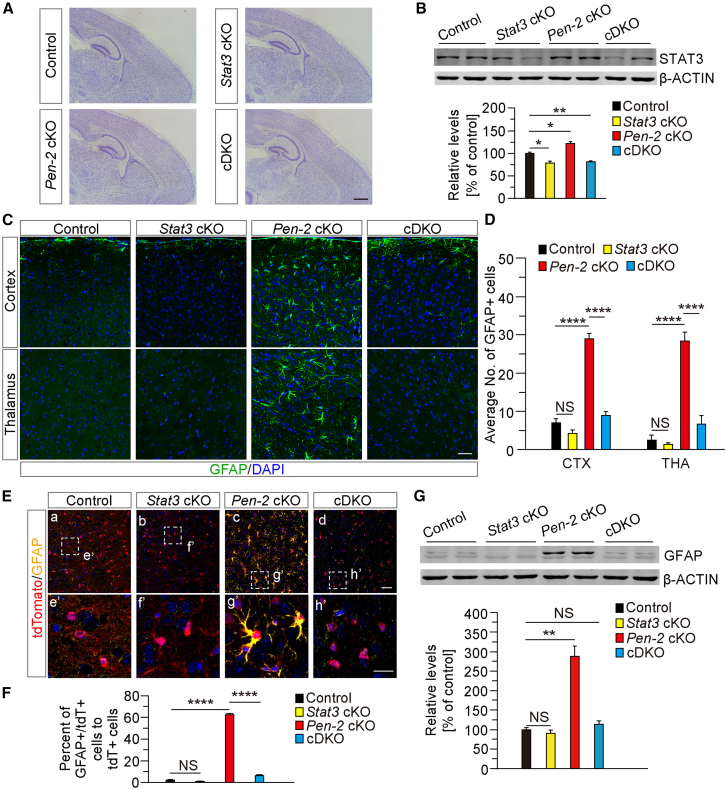
STAT3 deficiency restores astrocytes to normal levels in *Pen-2* cKO mice (A) Representative images of Nissl staining. Brain sections at P14 were prepared from four groups of mice: control, *Pen-2* cKO, *Stat3* cKO, and *Pen-2/Stat3* cDKO. No detectable changes in brain morphology were observed in *Pen-2/Stat3* cDKO mice compared with controls. Scale bars, 1 mm. (B) Western blotting analysis of STAT3. Cortical samples from P14 mice were used. STAT3 levels were significantly reduced in *Stat3* cKO and *Pen-2/Stat3* cDKO mice compared with controls (n = 3–4 mice per group; ^∗^*p* < 0.05; ^∗∗^*p* < 0.01). (C) Representative fluorescence IHC images for GFAP. Brain sections at P14 were used. Immunoreactivity of GFAP was reduced in *Pen-2/Stat3* cDKO mice compared with *Pen-2* cKO littermates. Scale bars, 50 μm. (D) Quantification of the average number of GFAP+ cells in the cortex and thalamus. A significant difference was observed between *Pen-2* cKO and *Pen-2/Stat3* cDKO mice (n = 3–6 mice per group; ^∗∗^*p* < 0.01; NS, not significant). (E) Representative images of co-staining for tdTomato and GFAP. Brain sections from tdTomato-expressing mice were used. The boxed areas in (a–d) are enlarged in (e’–h’). Scale bars, 50 μm in (a–d) or 25 μm in (e’–h’). (F) Quantification of the ratio of GFAP+/tdTomato+ cells to tdTomato+ cells (%). Cells in the cortex were counted using brain sections from P30 mice (n = 5–8 mice per group; ^∗∗∗∗^*p* < 0.0001; NS, not significant). (G) Western blotting analysis of GFAP. Cortical samples from four groups of P14 mice were used. Relative levels of GFAP were significantly decreased in *Pen-2/Stat3* cDKO mice compared with *Pen-2* cKO mice (n = 3–4 mice per group; ^∗∗^*p* < 0.01; NS, not significant).

Since STAT3 is known to play a critical role in the proliferation and differentiation of NG2 cells (Hackett et al., 2016; Steelman et al., 2016), we next investigated whether STAT3 is a key factor responsible for the changed glial populations in *Pen-2* cKO mice. We took advantage of floxed *Stat3* mice (Moh et al., 2007) to generate *Pen-2/Stat3* double cKO (cDKO) (*Pen-2*^*f/f*^*;Stat3*^*f/f*^*;Olig1-Cre*) mice (Figure S2A). Nissl staining revealed comparable brain morphology among *Pen-2* cKO, *Stat3* cKO, and *Pen-2/Stat3* cDKO mice and littermate controls at P14 (Figure 2A). Western blotting demonstrated significantly decreased levels of STAT3 in *Stat3* cKO and *Pen-2/Stat3* cDKO mice compared with age-matched controls (Figure 2B). While levels of APP C-terminal fragment (APP CTF) were significantly increased in *Pen-2* cKO and *Pen-2/Stat3* cDKO mice compared with controls, those of APP full-length (APP FL) remained unchanged (Figures S2B and S2C), confirming impaired γ-secretase activity.

To examine the effect of STAT3 deficiency on astrocytes in *Pen-2* cKO mice, we first performed fluorescence IHC for GFAP using brain sections at P14 (Figure 2C). The average number of GFAP+ cells in the cortex was significantly reduced in *Pen-2/Stat3* cDKO mice compared with *Pen-2* cKOs, but no significant difference was observed between control and *Pen-2/Stat3* cDKO mice (Figure 2D). Second, we conducted a lineage-tracing experiment. A breeding strategy was used to generate four groups of mice, including control, *Pen-2* cKO, *Stat3* cKO, and *Pen-2/Stat3* cDKO, expressing tdTomato in a Cre-dependent manner (Figure S2D). Double staining revealed that the average number of GFAP+/tdTomato+ cells in the cortex was significantly reduced in *Pen-2/Stat3* cDKO mice compared with *Pen-2* cKO littermates, but no significant difference was observed between control and *Pen-2/Stat3* cDKO mice (Figures 2E and 2F). Additionally, GS immunostaining showed that the average number of GS+ cells was significantly decreased in *Pen-2/Stat3* cDKO mice compared to *Pen-2* cKOs but did not differ between control and *Pen-2/Stat3* cDKO mice (Figures S2E and S2F). Western blotting further demonstrated that cortical levels of GFAP were significantly decreased in *Pen-2/Stat3* cDKO mice compared with *Pen-2* cKOs, while no significant difference was detected between control and *Pen-2/Stat3* cDKO mice (Figure 2G). Overall, these results suggest that inactivation of STAT3 restores the astrocyte population in *Pen-2* cKO mice. Therefore, Stat3 may mediate the OPC-to-astrocyte transdifferentiation in *Pen-2* cKO mice.

**Figure 3:**
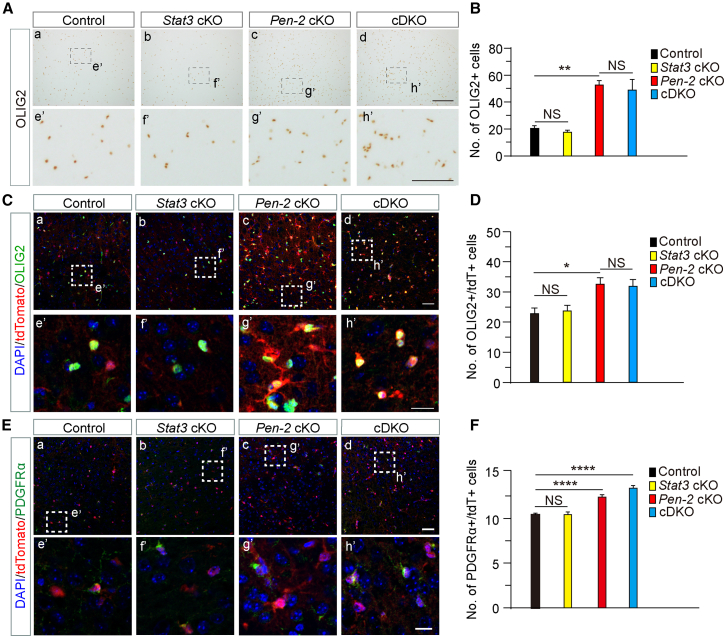
STAT3 deficiency fails to rescue the aberrant OPC population in *Pen-2* cKO mice (A) Representative IHC images for OLIG2. Brain sections from four groups of mice at P14 were used. Immunoreactivity of OLIG2 was comparable between *Pen-2* cKO and *Pen-2/Stat3* cDKO mice. The boxed areas in (a–d) are enlarged in (e’–h’). Scale bars, 50 μm in (a–d) or 25 μm in (e’–h’). (B) Quantification of the average number of OLIG2+ cells in the cortex. No significant difference was observed between *Pen-2* cKO and *Pen-2/Stat3* cDKO mice (n = 3–6 mice per group; ^∗∗^*p* < 0.01; NS, not significant). (C) Representative images of co-staining for OLIG2 and tdTomato. Brain sections at P30 were used. The boxed areas in (a–d) are enlarged in (e’–h’). Scale bars, 50 μm in (a–d) or 25 μm in (e’–h’). (D) Quantification of the average number of OLIG2+/tdTomato+ cells in the cortex. No significant difference was observed between *Pen-2* cKO and *Pen-2/Stat3* cDKO mice (n = 5–8 mice per group; ^∗^*p* < 0.05; NS, not significant). (E) Representative fluorescence images of co-staining for PDGFRα and tdTomato. Brain sections from mice at P30 were used. The boxed areas in (a–d) are enlarged in (e’–h’). Increased immunoreactivity of Pdgfrα was observed in *Pen-2* cKO and *Pen-2/Stat3* cDKO mice compared with controls. Scale bars, 50 μm in (a–d) or 25 μm in (e’–h’). (F) Quantification of the average number of PDGFRα+/tdTomato+ cells in the cortex. Cells were counted and averaged across sections. Significant differences were observed between control and *Pen-2* cKO or *Pen-2/Stat3* cDKO mice (n = 5–8 mice per group; ^∗∗∗∗^*p* < 0.0001; NS, not significant).

To assess the impact of STAT3 deficiency on OPCs in *Pen-2* cKO mice, we performed morphological and biochemical analyses. Although the average number of OLIG2+ cells in the cortex did not differ between *Pen-2* cKO and *Pen-2/Stat3* cDKO mice, it was significantly higher in the *Pen-2/Stat3* cDKO mice group compared to littermate controls (Figures 3A and 3B). Next, *Pen-2* cKO, *Stat3* cKO, and *Pen-2/Stat3* cDKO mice expressing tdTomato were used for lineage-tracing experiments (Figure S2D). Double staining revealed no significant difference in the number of OLIG2+/tdTomato+ cells in the cortex between *Pen-2* cKO and *Pen-2/Stat3* cDKO mice (Figures 3C and 3D). Similarly, the number of PDGFRα+/tdTomato+ cells did not differ significantly between the two groups (Figures 3E and 3F). Western blot analysis further confirmed that cortical OLIG2 protein levels were similar between *Pen-2* cKO and *Pen-2/Stat3* cDKO mice (Figure S3). Collectively, these results indicate that the OPC population was not restored to normal levels in *Pen-2/Stat3* cDKO mice, suggesting that STAT3 is unlikely to mediate the expansion of OPCs observed in *Pen-2* cKO mice.

### Mechanistic analysis to search for targets mediating Pen-2-dependent OPC self-renewal

**Figure 4:**
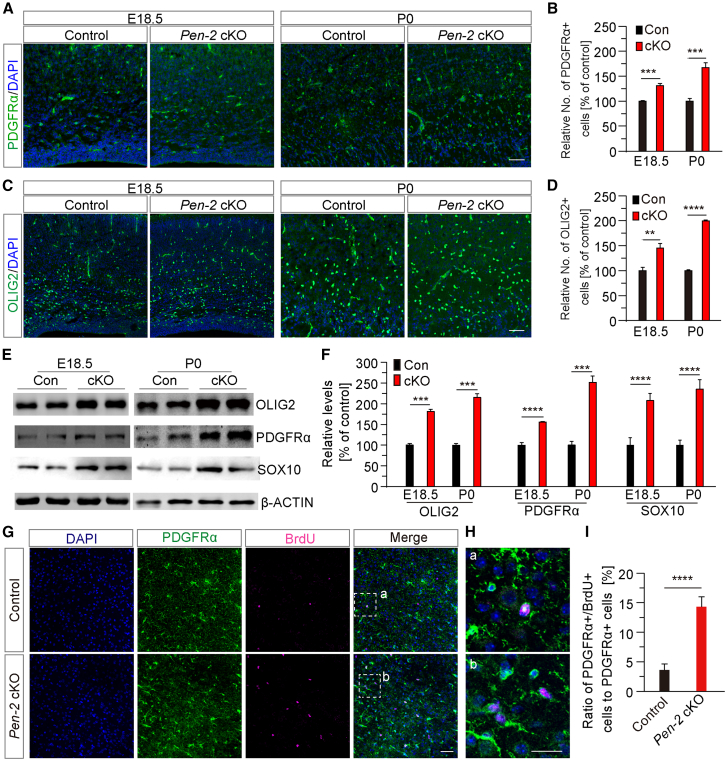
The expansion of OPCs is observed in *Pen-2* cKO mice at early developmental stages (A) Representative fluorescence IHC images for PDGFRα. Brain sections from control and *Pen-2* cKO mice at E18.5 and P0 were used. Increased immunoreactivity of PDGFRα was observed in *Pen-2* cKO mice compared with controls. Scale bars, 50 μm. (B) Quantification of the relative number of PDGFRα+ cells in *Pen-2* cKO mice (% of controls). Cells in the cortex were counted using sections at E18.5 and P0. Significant differences in the average number of PDGFRα+ cells were observed between control and *Pen-2* cKO mice at E18.5 and P0 (control: n = 5–6; *Pen-2* cKO: n = 4–6; ^∗∗∗^*p* < 0.001). (C) Representative fluorescence IHC images for OLIG2. Increased immunoreactivity of OLIG2 was observed in *Pen-2* cKO mice compared with controls at E18.5 and P0. Scale bars, 50 μm. (D) Quantification of the relative number of OLIG2+ cells in *Pen-2* cKO mice (% of controls). Significant differences in the average number of OLIG2+ cells were observed between control and *Pen-2* cKO mice at E18.5 and P0 (control: n = 4–5; *Pen-2* cKO: n = 3–4; ^∗∗^*p* < 0.01; ^∗∗∗∗^*p* < 0.0001). (E and F) Western blotting analysis of OLIG2, SOX10, and PDGFRα. Cortical samples from mice at E18.5 and P0 were used. Relative levels of OLIG2, SOX10, and PDGFRα were significantly increased in *Pen-2* cKO mice compared with controls (control: *n* = 3; *Pen-2* cKO: *n* = 4; ^∗∗∗^*p* < 0.001; ^∗∗∗∗^*p* < 0.0001). β-ACTIN and GAPDH served as the loading control. (G and H) Representative fluorescence images of co-staining of PDGFRα and BrdU. Brain sections from mice at P0 were used. The boxed areas in (G) are enlarged in (H). Scale bars, 50 μm in (G) or 25 μm in (H). (I) Quantification of the ratio of PDGFRα+/BrdU+ cells to PDGFRα+ cells (%). Cells in the cortex were counted. A significant difference was observed between control and *Pen-2* cKO mice (*n* = 5 per group; ^∗∗∗∗^*p* < 0.0001).

To further explore the molecular mechanisms underlying the expanded OPC population in *Pen-2* cKO mice, we examined when this phenotype first emerged. We performed fluorescence IHC for PDGFRα and OLIG2 using brain sections from mice at various developmental stages, including P0, embryonic day 18.5 (E18.5), and E16.5 (Figures 4A–4C and S4A). At E18.5 and P0, *Pen-2* cKO mice exhibited a significant increase in the average number of PDGFRα+ and OLIG2+ cells compared to littermate controls (Figures 4B and 4D), indicating that OPC expansion occurs during late embryogenesis. In contrast, no significant difference in OLIG2+ cell number was observed between control and *Pen-2* cKO mice at E16.5 across different brain subregions (Figures S4A and S4B). Additionally, while cortical levels of PDGFRα, OLIG2, and SOX10 were similar between genotypes at E17.5 (Figures S4C and S4D), they were significantly elevated in *Pen-2* cKO mice at E18.5 and P0 (Figures 4E and 4F). These findings suggest that the OPC expansion in *Pen-2* cKO mice begins between E17.5 and E18.5, implicating a critical developmental window for this phenotype.

To assess the self-renewal of OPCs in *Pen-2* cKO mice, we conducted BrdU-pulse-labeling experiments using mice at P0. BrdU was intraperitoneally injected into the mice, and brain samples were collected 30 min post-injection. Co-staining of PDGFRα/BrdU revealed abundance of BrdU+ cells in *Pen-2* cKO mice compared with age-matched controls (Figures 4G and 4H). We observed that the ratio of PDGFRα+/BrdU+ cells to PDGFRα+ cells in the cortex was significantly increased in *Pen-2* cKO mice compared with littermate controls (Figure 4I). These results demonstrated that the expanded OPC population in *Pen-2* cKO mice is attributable to enhanced self-renewal of OPCs.

**Figure 5:**
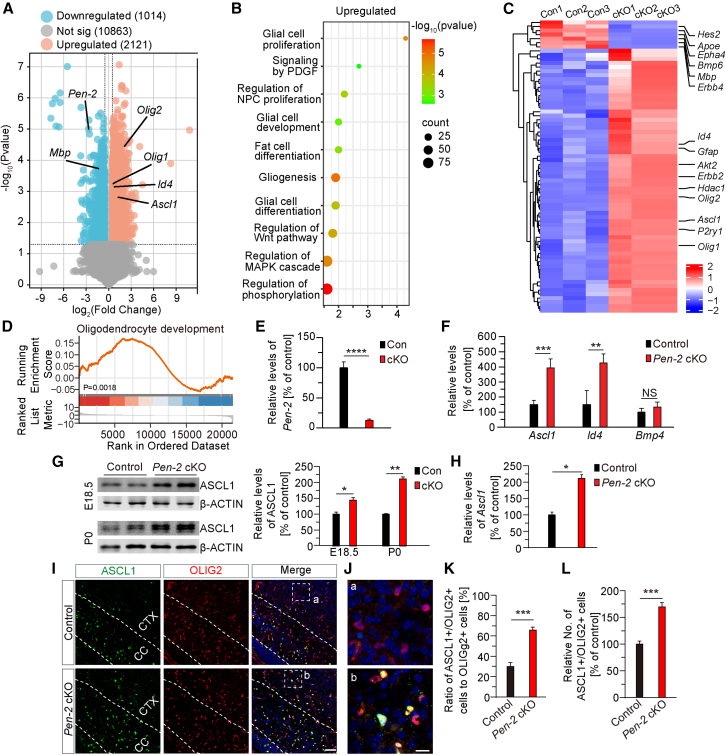
Upregulation of ASCL1 in Pen-2-deficient OL lineage cells (A) Volcano plot of DEGs between control and *Pen-2* cKO OPCs. Red and blue points represent genes with significantly increased or decreased mRNA levels, respectively (*p* < 0.05 and |log2 (foldchange)| > 0.5). (B) GO analysis for control and *Pen-2* cKO OPCs showing enriched biological processes for upregulated genes (*p* < 0.05, log_2_(fold change) > 0.5). (C) Heatmap analysis depicting RNA-seq results, with the red and blue indicating upregulated and downregulated mRNAs, respectively. A number of DEGs critical for OPC development and OL differentiation were identified in *Pen-2* cKO OPCs compared with controls. (D) Gene set enrichment analysis (GSEA) revealed a significant enrichment of oligodendrocyte-development-related genes in *Pen-2* cKO OPCs. (E and F) RT-qPCR analysis on mRNA levels of *Pen-2*, *Ascl1*, *Id4*, and *Bmp4* using RNA samples prepared from the cultured OPCs. Significant reduction in *Pen-2* mRNA levels was observed in cultured *Pen-2* cKO OPCs compared with controls (E). Significant increases in mRNA levels of *Ascl1* and *Id4* were observed in cultured *Pen-2* cKO OPCs compared with controls (F). No significant difference in *Bmp4* mRNA levels was observed between control and *Pen-2* cKO mice at P14 (F) (*n* = 4 per group; ^∗∗^*p* < 0.01; ^∗∗∗^*p* < 0.001; ^∗∗∗∗^*p* < 0.0001; NS, not significant). (G) Western blot analysis of ASCL1 using cortical samples from control and *Pen-2* cKO mice at E18.5 and P0. Significant difference in ASCL1 protein levels were observed between control and *Pen-2* cKO mice at both ages (control: n = 3–4; cKO: n = 4–5; ^∗^*p* < 0.05; ^∗∗^*p* < 0.01). (H) RT-qPCR analysis on mRNA levels of *Ascl1* using cortical samples from control and *Pen-2* cKO mice at E18.5. Relative mRNA levels of *Ascl1* were significantly increased in *Pen-2* cKO mice at E18.5 compared with controls (*n* = 4 per group; ^∗^*p* < 0.05). (I and J) Representative fluorescence images of co-staining for ASCL1 and OLIG2. Brain sections at P0 were used. The boxed areas in (I) are enlarged in (J). Scale bars, 50 μm in (I) or 25 μm in (J). (K) Quantification of the ratio of ASCL1+/OLIG2+ cells to OLIG2+ cells (%). Cells in the cortex were counted. A significant difference was observed between control and *Pen-2* cKO mice (*n* = 8 per group; ^∗∗∗^*p* < 0.001). (L) Quantification of the relative number of ASCL1+/OLIG2+ cells in *Pen-2* cKO cortices (% of controls). A significant difference was observed between the two genotype groups (*n* = 8 per group; ^∗∗∗^*p* < 0.001).

To search for the molecular mechanisms underlying the altered OPC population in *Pen-2* cKO mice, we performed RNA sequencing (RNA-seq) analysis using RNA samples isolated from cultured primary OPCs derived from cortical tissues of mice at P8. Principal-component analysis (PCA) revealed clear segregation of the two genotype groups based on principal components 1 and 2 (PC1/PC2) (Figure S5A). The volcano plot identified more than 3,000 differentially expressed genes (DEGs) in *Pen-2* cKO OPCs compared with controls (Figure 5A). As expected, *Pen-2* and *Mbp* were significantly downregulated in *Pen-2* cKO OPCs (Figure 5A). Among the significantly upregulated genes, *Olig1*, *Olig2*, and *Id4* are well-known regulators of OPC fate determination (Figure 5A). Gene Ontology (GO) analysis indicated that upregulated DEGs were enriched in several signaling pathways, including glial cell proliferation, glial cell development, and gliogenesis (Figure 5B), while downregulated DEGs were enriched in pathways related to the regulation of nervous development in cultured *Pen-2* cKO OPCs (Figure S5B). Based on these transcriptomic results, a heatmap was generated to display the expression profiles of genes associated with glial cell development (Figure 5C). We observed elevated expression levels of numerous genes critical for OPC proliferation in *Pen-2* cKO OPCs compared with control OPCs (Figure 5C). Furthermore, gene set enrichment analysis (GSEA) revealed significant enrichment of genes essential for OL development in *Pen-2* cKO OPCs (Figure 5D). Interestingly, GSEA also indicated significant reductions in genes involved in cholesterol and steroid metabolism in *Pen-2* cKO OPCs compared with controls (Figure S5C). Indeed, a large number of genes associated with cholesterol and steroid biosynthesis were significantly downregulated in *Pen-2* cKO OPCs compared with controls (Figure S5D). Collectively, these findings suggest that Pen-2 deficiency disrupts cholesterol and steroid metabolism in OL lineage cells, consistent with a recent study demonstrating that chronic γ-secretase suppression markedly reduces cholesterol levels in neurons (Essayan-Perez and Sudhof, 2023).

To validate the transcriptomic results, we selected *Ascl1*, *Id4*, and *Bmp4* for quantitative real-time PCR analysis, as these molecules are known to play critical roles in neurogenesis, gliogenesis, and astrogliogenesis. First, our quantitative real-time PCR data confirmed a significant reduction on *Pen-2* mRNA levels in *Pen-2* cKO OPC cultures compared with controls (Figure 5E). Second, mRNA levels of *Ascl1* and *Id4*, but not *Bmp4*, were significantly elevated in *Pen-2* cKO OPC cultures (Figure 5F). Overall, the quantitative real-time PCR results were consistent with the RNA-seq findings.

### ASCL1 mediates the enhanced self-renewal of Pen-2-deficient OPCs

Given the critical role of ASCL1 in gliogenesis (Vue et al., 2014), we conducted the following experiments to validate its involvement in Pen-2-dependent OPC self-renewal. First, we performed biochemical and IHC analyses using brain samples from mice at E18.5 and P0. Western blotting revealed significantly elevated levels of ASCL1 in *Pen-2* cKO mice at E18.5 and P0 compared with littermate controls (Figure 5G). Our quantitative real-time PCR data further confirmed increased levels of *Ascl1* mRNAs in *Pen-2* cKO cortices at P0 (Figure 5H). Third, co-staining of ASCL1 and OLIG2 demonstrated qualitatively enhanced immunoreactivity of ASCL1 in *Pen-2* cKO cortices compared with littermate controls at P0 (Figures 5I and 5J). Fourth, we observed a significantly higher ratio of OLIG2+/ASCL1+ cells to OLIG2+ cells in the cortex in *Pen-2* cKO mice than in littermate controls (Figure 5K). Finally, the relative number of ASCL1+/OLIG2+ cells in the cortex was significantly greater in *Pen-2* cKO cortices than in littermate controls (Figure 5L). Collectively, these findings suggest that upregulation of ASCL1 is associated with enhanced OPC self-renewal in *Pen-2* cKO mice.

**Figure 6:**
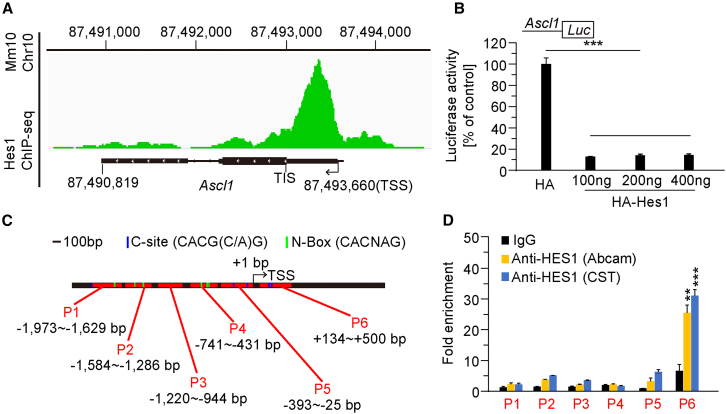
Pen-2 regulates *Ascl1* expression via HES1 (A) Re-analysis of Hes1 ChIP-seq data from Shang et al. (2016). A Hes1-binding domain was identified within the *Ascl1* promoter. (B) Luciferase assay of relative *Ascl1* promoter activity and its concentration-dependent inhibition by HES1 overexpression in HEK293T cells. Significant reductions in *Ascl1* promoter activity were observed in cells transfected with different doses of Hes1 compared with cells transfected with the control luciferase reporter (*n* = 3 independent experiments; ^∗∗∗^*p* < 0.001). (C) Primer design for six regions (P1–P6) in the *Ascl1* promoter. Specific primers targeting P1–P6 were used for ChIP-qPCR analysis. (D) ChIP-qPCR analysis of HES1 binding to the *Ascl1* promoter region in OPCs. Data are expressed as fold enrichment relative to the corresponding immunoglobulin G (IgG) control (^∗∗^*p* < 0.01; ^∗∗∗^*p* < 0.001). Significant enrichment was observed for the P6 primer pair using two different HES1 antibodies compared with the IgG control. Results are shown from three independent experiments.

Next, we re-analyzed data from a previous chromatin immunoprecipitation (ChIP) study on Hes1 (Shang et al., 2016). We identified an interaction domain within the promoter region of the *Ascl1* gene for HES1 (Figure 6A). In contrast, the abovementioned analysis did not reveal HES1-binding domain in the promoter of *Id4*. In addition, BMP4 expression was unchanged in *Pen-2* cKO mice (Figures 5A and 5F). Thus, it is likely that ID4 and BMP4 are not direct targets of HES1. For these reasons, we focused on testing the hypothesis that HES1 may regulate *Ascl1* expression at the transcriptional level. First, we carried out a series of luciferase assays using cultured HEK293T cells. Plasmids expressing HES1 and a luciferase reporter driven by the *Ascl1* promoter were co-transfected. Our results demonstrated that the expression of HES1 at three different doses robustly suppressed the promoter activity of *Ascl1* (Figure 6B). Second, to identify the Hes1-binding region within the *Ascl1* promoter, we performed ChIP-qPCR analysis. Six pairs of primers were designed to cover subregions of the *Ascl1* promoter (Figure 6C). Immunoprecipitation was conducted using two anti-HES1 antibodies and samples of primary OPCs cultured from cortical tissues of control and *Pen-2* cKO mice at P8. Using the first anti-HES1 antibody, qPCR results revealed significant enrichment for the primer pair covering the region from +134 bp to +500 bp. In contrast, no significant enrichment was observed for other five primer pairs, which covered promoter regions from −1973 to −1629 bp, −1584 to −1286 bp, −1220 to −944 bp, −741 to −431 bp, or −393 to −25 bp (Figure 6D). Similar results were obtained with the second anti-HES1 antibody (Figure 6D). Third, we conducted an *in vivo* experiment to validate the role of the Notch/Hes1 signaling in ASCL1 expression. *Pen-2*^*f/+*^*;Olig1-Cre* mice were crossed to *N1ICD* transgenics (Cheng et al., 2019) to generate *Pen-2* cKO mice expressing NICD (*Pen-2*^*f/f*^*;Olig1-Cre;LSL-N1ICD*) (Figure S6A). We performed western blotting for Pen-2, NICD, and ASCL1 using cortical samples prepared from mice aged at P0 (Figure S6B). Cortical levels of NICD were significantly decreased in *Pen-2* cKO mice compared with littermate controls, whereas they were significantly increased in *Pen-2* cKO;*N1ICD* mice compared with *Pen-2* cKOs (Figure S6C). We observed that ASCL1 levels were significantly elevated in the cortex of *Pen-2* cKO mice compared with littermate controls, while they were significantly reduced in *Pen-2* cKO;*N1ICD* mice compared with *Pen-2* cKOs (Figures S6B and S6C). Furthermore, to find out whether HES1 expression was affected, we performed immunostaining and quantitative real-time PCR analyses. While HES1 fluorescence intensity and *Hes1* mRNA levels in the cortex were comparable between control and *Pen-2* cKO;*N1ICD* mice, they were significantly decreased in *Pen-2* cKO mice (Figures S6E–S6G). Collectively, the above *in vitro* and *in vivo* results suggest that Pen-2 may regulate *Ascl1* expression through HES1.

**Figure 7:**
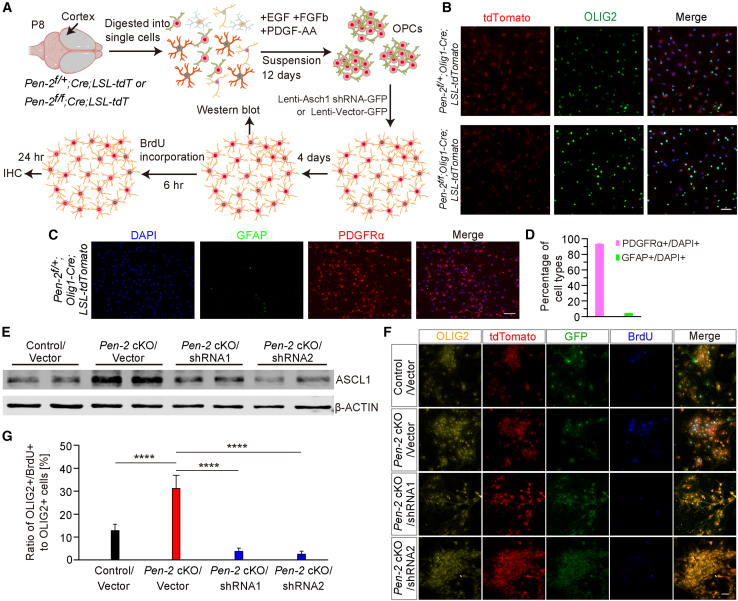
Knockdown of *Ascl1* restores the self-renewal capacity of Pen-2-deficient OPCs (A) Experimental design for an *in vitro* study. Primary OPCs were cultured from cortical tissues of *Pen-2*^*f/+*^*;Olig1-Cre;LSL-tdTomato* and *Pen-2*^*f/f*^*;Olig1-Cre;LSL-tdTomato* mice at P8. Two sets of short hairpin RNA (shRNA) targeting Ascl1 were designed for transfection. Cell samples were collected at different time points as indicated. (B and C) Representative fluorescence images of co-staining for tdTomato and OLIG2 in primary OPC cultures. (C and D) Triple-immunostaining for PDGFRα, GFAP, and DAPI revealed that the vast majority of DAPI+ cells in primary OPC cultures were PDGFRα+, while only a very small fraction were GFAP+. (E) Western blotting analysis of ASCL1. Cell lysates were collected from four groups: control OPCs, *Pen-2* cKO OPCs, and *Pen-2* cKO OPCs infected with either *Ascl1*-shRNA1 or *Ascl1*-shRNA2. ASCL1 levels were reduced in *Pen-2* cKO OPCs infected with *Ascl1*-shRNA1 and *Ascl1*-shRNA2 compared with *Pen-2* cKO OPCs. (F) Representative fluorescence images of co-staining of OLIG2/tdTomato/GFP/BrdU. Primary OPC cultures were transfected with plasmids expressing control GFP, *Ascl1*-shRNA1-GFP, or *Ascl1*-shRNA2-GFP. Numerous OLIG2+/tdTomato+/GFP+/BrdU+ cells were observed in *Pen-2* cKO OPCs expressing control GFP. (G) The ratio of OLIG2+/tdTomato+ cells to OLIG2+ cells (%). Primary OPCs were cultured from three *Pen-2* cKO mice expressing tdTomato and were transfected with control GFP, *Ascl1*-shRNA1-GFP, or *Ascl1*-shRNA2-GFP plasmids, respectively. A significant reduction was observed in the percentage of OLIG2+/tdTomato+ cells in cultures expressing *Ascl1*-shRNA1-GFP or *Ascl1*-shRNA2-GFP compared to the control GFP group (*n* = 3 mice per group; ^∗∗∗∗^*p* < 0.0001).

To validate the role of ASCL1 in the self-renewal of *Pen-2* cKO OPCs, we conducted a rescue experiment using primary OPC cultures (Figure 7). OPCs were isolated from the cortices of control (*Pen-2*^*f/+*^*;Olig1-Cre*) and *Pen-2* cKO (*Pen-2*^*f/f*^*;Olig1-Cre*) mice expressing tdTomato (Figures 7A and 7B). We evaluated the purity of our OPC cultures by performing immunocytochemical analysis for PDGFRα and GFAP. Quantification showed that ∼93% of DAPI+ cells were PDGFRα+, while ∼4% were GFAP+, demonstrating high OPC purity with minimal astrocyte contamination (Figures 7C and 7D). Cultured cells were transduced with lentiviral vectors encoding GFP, *Ascl1*-shRNA1-GFP, or *Ascl1*-shRNA2-GFP. Western blot analysis revealed significantly elevated ASCL1 levels in *Pen-2* cKO OPCs infected with GFP compared to control OPCs. However, ASCL1 expression was markedly reduced in Pen-2 cKO OPCs following infection with either *Ascl1*-shRNA1-GFP or *Ascl1*-shRNA2-GFP (Figure 7E), confirming efficient knockdown.

To assess the effect of *Ascl1* knockdown on OPC self-renewal, BrdU-pulse-labeling was performed by supplementing the culture medium with BrdU for 6 h. Co-staining for OLIG2, BrdU, GFP, and tdTomato showed a reduction in BrdU+ cells among *Pen-2* cKO OPCs treated with *Ascl1*-shRNA1-GFP or *Ascl1*-shRNA2-GFP (Figure 7F). Quantification revealed a significantly lower ratio of OLIG2+/BrdU+ cells to total OLIG2+ cells in the *Ascl1*-shRNA-treated groups compared to the GFP control (Figure 7G). Notably, Pen-2 cKO OPCs exhibited a higher number of OLIG2+/BrdU+ cells than control OPCs (Figure 7G). Together, these findings demonstrate that *Ascl1* knockdown reduces the enhanced self-renewal capacity of *Pen-2* cKO OPCs, underscoring a key role for ASCL1 in this process.

## Discussion

Given the implication of PEN-2 in neurological diseases associated with abnormal glial populations, this study aimed to investigate the molecular mechanisms by which oligodendrocytic Pen-2 regulates glial homeostasis. Through a combination of *in vivo* and *in vitro* approaches, we uncovered several key findings. First, similar phenotypes observed in *Pen-2* cKO and *Ncstn* cKO mice suggest that Pen-2 regulates glial homeostasis in a γ-secretase-dependent manner. Second, phenotypic analysis of *Pen-2*/*Stat3* cDKO mice indicates that STAT3 mediates the expansion of astrocytes, but not OPCs, in *Pen-2* cKO mice. Third, transcriptomic and molecular analyses identify ASCL1 as a key mediator of OPC expansion in the absence of Pen-2. Collectively, these results demonstrate that Pen-2 orchestrates both OPC self-renewal and transdifferentiation programs to maintain glial homeostasis.

Although Pen-2 is primarily recognized as an essential component of the γ-secretase complex (Steiner et al., 2002), emerging evidence also supports a γ-secretase-independent role (Ma et al., 2022). One of our objectives was to test whether Pen-2 regulates glial population homeostasis specifically via γ-secretase activity. Since NCSTN has not been shown to function independently of γ-secretase, we used two lines of OL-lineage-specific knockout mice, *Pen-2* cKO and *Ncstn* cKO, for direct comparison. The finding that both models exhibit increased numbers of astrocytes and OPCs strongly supports a γ-secretase-dependent mechanism. Interestingly, previous work has shown that deletion of PSEN1, another γ-secretase component, results in stage-specific enhancement of astrogliogenesis in the mouse cortex (Sardi et al., 2006). Our findings here demonstrate that deletion of distinct γ-secretase subunits disrupts the balance of astrocyte, OPC, and OL populations. These results suggest that oligodendrocytic Pen-2 concurrently regulates astrogliogenesis and oligodendrogenesis in a γ-secretase-dependent fashion. Notably, other oligodendrocytic TFs, including OLIG2, HDAC3, and RBPj, also play an important role in astrogliogenesis. For example, inactivation of OLIG2 or HDAC3 leads to decreased OPC population and increased astrocyte population in the cortex (Zhu et al., 2012; Zhang et al., 2016). HDAC3 appears to act upstream of OLIG2, modulating its interaction with p300 to suppress astrocyte differentiation (Zhang et al., 2016). Additionally, RBPj has been shown to influence astrogliogenesis through the BMP4 signaling in premyelinating OLs (Guo et al., 2023). Interestingly, our analyses revealed that the expression levels of HDAC3 and BMP4 remain unchanged in *Pen-2* cKO mice, suggesting that these molecules are not involved in Pen-2-mediated astrogliogenesis.

In our previous study, we reported that Olig1-Cre- or NG2-CreERT2-mediated deletion of Pen-2 leads to an expansion of both astrocytes and OPCs, accompanied by elevated STAT3 expression (Hou et al., 2021). Given the established role of STAT3 in NG2 cell proliferation (Hackett et al., 2016; Steelman et al., 2016), we initially hypothesized that STAT3 might be a key mediator of these phenotypes in *Pen-2* cKO mice. Interestingly, the STAT3 rescue experiments showed that STAT3 inactivation completely restored the astrocyte population but did not normalize the OPC population in *Pen-2* cKO mice. This unexpected divergence prompted us to investigate alternative mechanisms underlying the OPC phenotype. Through comprehensive transcriptomic, biochemical, molecular, and cellular analyses, we identified ASCL1 as a critical regulator of Pen-2-dependent OPC self-renewal. Several lines of evidence support this conclusion. First, prior studies have shown that ASCL1 is essential for gliogenesis (Vue et al., 2014), and its inactivation leads to reduced OPC numbers in the spinal cord (Sugimori et al., 2008). Second, we observed increased ASCL1 expression in OLIG2+ cells in the *Pen-2* cKO cortex and in cultured *Pen-2* cKO OPCs. We further demonstrated that Pen-2 regulates *Ascl1* expression through HES1. Third, we identified a critical *Hes1*-binding site within the *Ascl1* promoter. Most importantly, *Ascl1* knockdown effectively restored the self-renewal capacity of cultured *Pen-2* cKO OPCs. Fourth, a recent study demonstrated the dual role of Notch signaling in regulating the neuron-to-oligodendrocyte switch in the developing forebrain (Tran et al., 2023). It further revealed that *Hes1* and *Hes5* knockdown upregulates ASCL1 (Tran et al., 2023). Overall, these findings were in line with ours showing HES1 downregulation and ASCL1 upregulation in OL-lineage-specific *Pen-2* cKO mice.

The 2016 *Science* study has revealed the heterogeneity of OPCs, including differentiation-committed OPCs and PDGFRα+ cells associated with the vasculature and leptomeninges (Marques et al., 2016). In this study, while we observed an overall increase in the OPC population in the cortex of *Pen-2* cKO mice, we did not detect region-specific changes. Our molecular analyses suggest that Pen-2 regulates OPC self-renewal through the Notch/HES1/ASCL1 signaling pathway. Given that Notch/Hes signaling is a conserved mechanism in both neural progenitor cells and OPCs, we propose that Pen-2 deletion likely affects multiple OPC subtypes similarly. However, since we used PDGFRα, a general marker, to label OPCs, we cannot exclude the possibility that Pen-2 deficiency differentially impacts specific OPC subtypes. While this hypothesis could be further tested once subtype-specific antibodies become available, future single-cell transcriptomic studies will be crucial in elucidating the role of Pen-2 in distinct OPC subtypes in the CNS.

Collectively, our findings demonstrate that ASCL1 and STAT3 play distinct yet essential roles, ASCL1 in OPC self-renewal and STAT3 in OPC-to-astrocyte conversion, thereby revealing that Pen-2 maintains glial homeostasis by coordinately regulating these two programs. Importantly, while our previous work primarily characterized the cellular and phenotypic consequences of Pen-2 deletion in OL lineage cells (Hou et al., 2021), the present study elucidates the underlying molecular mechanisms, significantly advancing our understanding of Pen-2’s role in glial development.

## Methods

A more detailed version of this section is available in supplemental information.

### Animals

Detailed information for the generation of *Pen-2* cKO, *Pen-2/Stat3* cDKO, and *Ncstn* cKO mice was described in supplemental information. All animal experiments were approved by the Institutional Animal Care and Use Committee (IACUC) of MARC, Nanjing University, and conducted in accordance with the Guide to the Care and Use of Laboratory Animals of MARC, Nanjing University.

### Cell culture and plasmids transfection

HEK293T cells were cultured in DMEM (Invitrogen) supplemented with 10% fetal bovine serum (FBS, Gibco) and 1% penicillin/streptomycin solution at 37°C in a humidified incubator with 5% CO_2_. After 24 h of incubation, transient transfection was performed according to the manufacturer’s protocol for Lipofectamine 2000 transfection reagent. The medium was replaced 6 h post-transfection, and the cells were harvested at 48 h after transfection.

### Western blotting

Sample protein concentration was determined using a standard BSA method described previously (Hou et al., 2021). The antibodies used were listed in Table S1.

### Chromatin immunoprecipitation

OLs were fixed with 1% formaldehyde, and the cross-linking reaction was terminated by adding glycine. The cross-linked chromatin was then fragmented by sonication using a Bioruptor (Diagenode). The resulting chromatin fragments were incubated overnight at 4°C with specific antibodies against HES1 (Abcam, ab71559; Cell Signaling Technology, 11988; 2 μg each) or Normal Rabbit IgG (Cell Signaling Technology, 2729; 2 μg). Dynabeads Protein G was added, and the mixture was incubated with rotation for 6 h at 4°C. The beads were isolated and washed three times with low-salt buffer and once with high-salt buffer. Immune complexes were eluted from the beads, and the eluate was incubated overnight at 65°C. Proteins were digested with proteinase K at 45°C for 2 h, and the remaining DNA was purified using a spin column. The eluted DNA was analyzed by quantitative real-time PCR using an Applied Biosystems Prism StepOne Plus system with 2× RealStar Green Fast Mixture with Rox. Enrichment was calculated as 2^−ΔCt^, where ΔCt represents the difference in cycle threshold (Ct) values between ChIP and immunoglobulin G (IgG) samples. The amount of target genomic DNA was normalized to the input DNA. PCR primer information was listed in Table S2.

### Statistical analysis

Statistical analyses were performed using GraphPad Prism software version 6 (GraphPad Software, La Jolla, CA). Data are presented as mean ± standard error of the mean (SEM). Differences between two experimental groups were analyzed using a two-tailed Student’s t test, while comparisons among multiple groups were assessed using one-way analysis of variance (ANOVA). A *p* value of less than 0.05 was considered statistically significant. For western blotting, cell counting, quantitative real-time PCR, and RNA-seq experiments, at least three mice were used per group.

## Resource availability

### Lead contact

Requests for further information and resources should be directed to the lead contact, Guiquan Chen (chenguiquan@nju.edu.cn).

### Materials availability

All unique reagents generated in the current study are available and will be fulfilled by the lead contact.

### Data and code availability

The datasets generated during the current study are available in the Sequence Read Archive (SRA): PRJNA1167880.

## Acknowledgments

This work was supported by grants from the 10.13039/501100001809National Natural Science Foundation of China (32270871 to G.C. and 82201528 to J.H.), the 10.13039/501100004608Natural Science Foundation of Jiangsu Province (BK20220169 to J.H.), the Jiangsu Funding Program for Excellent Postdoctoral Talent (2022ZB705 to H.B.), and the 10.13039/501100012226Fundamental Research Funds for the Central Universities (021414380533 to G.C.).

## Author contributions

H.B. designed the research, performed the experiments, analyzed the data, and drafted the manuscript. J.H. designed the research, performed part of the experiments, and analyzed the data. J.H., Y.X., Z.W., and G.C. provided materials, designed the research, and revised the manuscript. W.S., C.G., Y.L., and R.W. performed part of the experiments. All authors have read and approved the final manuscript.

## Declaration of interests

The authors declare no competing interests.

## Supplemental information

Document S1. Figures S1–S6, Tables S1 and S2, and supplemental methods

Document S2. Article plus supplemental information
