# Supplementary material for: Pen-2 regulates glial homeostasis by coordinating self-renewal and transdifferentiation programs in oligodendrocyte precursor cells
Source: Stem Cell Reports. 2025 Aug 28;20(9):102612. doi: 10.1016/j.stemcr.2025.102612 (PMC12447332; doi:10.1016/j.stemcr.2025.102612)
Supplement: Document S1. Figures S1–S6, Tables S1 and S2, and supplemental methods [file mmc1.pdf]

Stem Cell Reports, Volume *20*

Supplemental Information

Pen-2 regulates glial homeostasis by coordinating self-renewal and transdifferentiation programs in oligodendrocyte precursor cells

Huiru Bi, Jinxing Hou, Wenkai Shao, Chenyi Ge, Yang Liu, Runmin Wang, Guiquan Chen, Yun Xu, and Zhiye Wang

Supplemental Figures

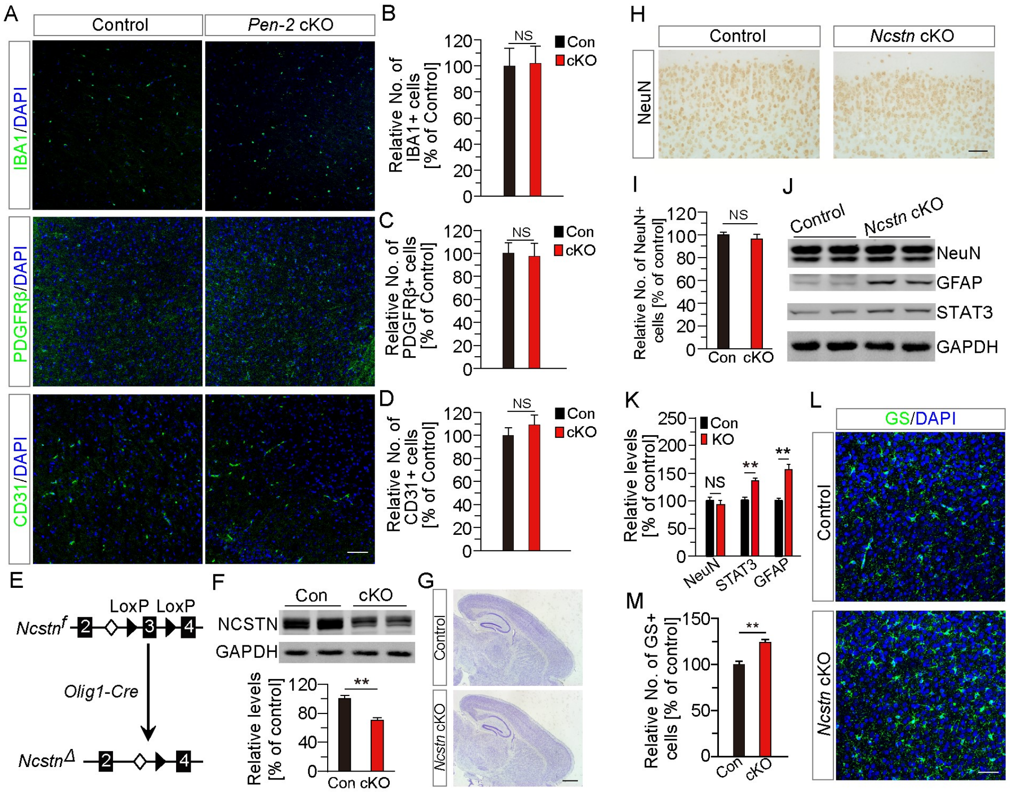

Fig. S1: Disrupted glial populations in OL lineage-specific *Ncstn* cKO mice.

A. Representative fluorescence IHC images for IBA1, PDGFRβ and CD31 in the cortex. Brain sections were prepared from mice at 4 months. The scale bar is 50 μm. **B-D.** Relative number of IBA1+, PDGFRβ+ and CD31+ cells [% of the control]. There was no significant difference between control and *Pen-2* cKO mice (Control: n = 3; *Pen-2* cKO: n = 3; NS, not significant). **E.** Schematic diagram for generating OL lineage-specific *Ncstn* cKO mice. **F.** Western blotting analysis of NCSTN. Cortical samples from control and *Ncstn* cKO mice at P14 were used. Levels of NCSTN were significantly reduced in *Ncstn* cKO mice compared with controls (n≥ 4 mice per group; **, $P < 0.01$). **G.** Representative images of Nissl staining. Brain sections from control and *Ncstn* cKO mice at P30 were used. No detectable changes in brain morphology were observed in *Ncstn* cKO mice compared with controls. The scale bar is 1 mm. **H.** Representative IHC images for NeuN. Immunoreactivity of NeuN was comparable between control and *Ncstn* cKO mice. The scale bar is 50 μm. **I.** Relative number of NeuN+ cells in the cortex. There was no significant difference between control and *Ncstn* cKO mice at P30 (n = 3 mice per group; NS, not significant). **J-K.** Western blotting analysis of NeuN, GFAP and STAT3. Cortical samples from control and *Ncstn* cKO mice at P30 were used (J).There was no significant difference in NeuN levels between control and *Ncstn* cKO mice at P30 (K). Protein levels of GFAP and STAT3 were significantly elevated in *Ncstn* cKO mice compared with controls (K) (n = 4 mice per group; **, $P < 0.01$; NS, not significant). **L-M.** Fluorescence IHC showing significantly increased number of GS+ cells in *Ncstn* cKO mice at P30 (Control: n = 4; *Ncstn* cKO: n = 4; **, P <0.01). The scale bar is 50 μm.

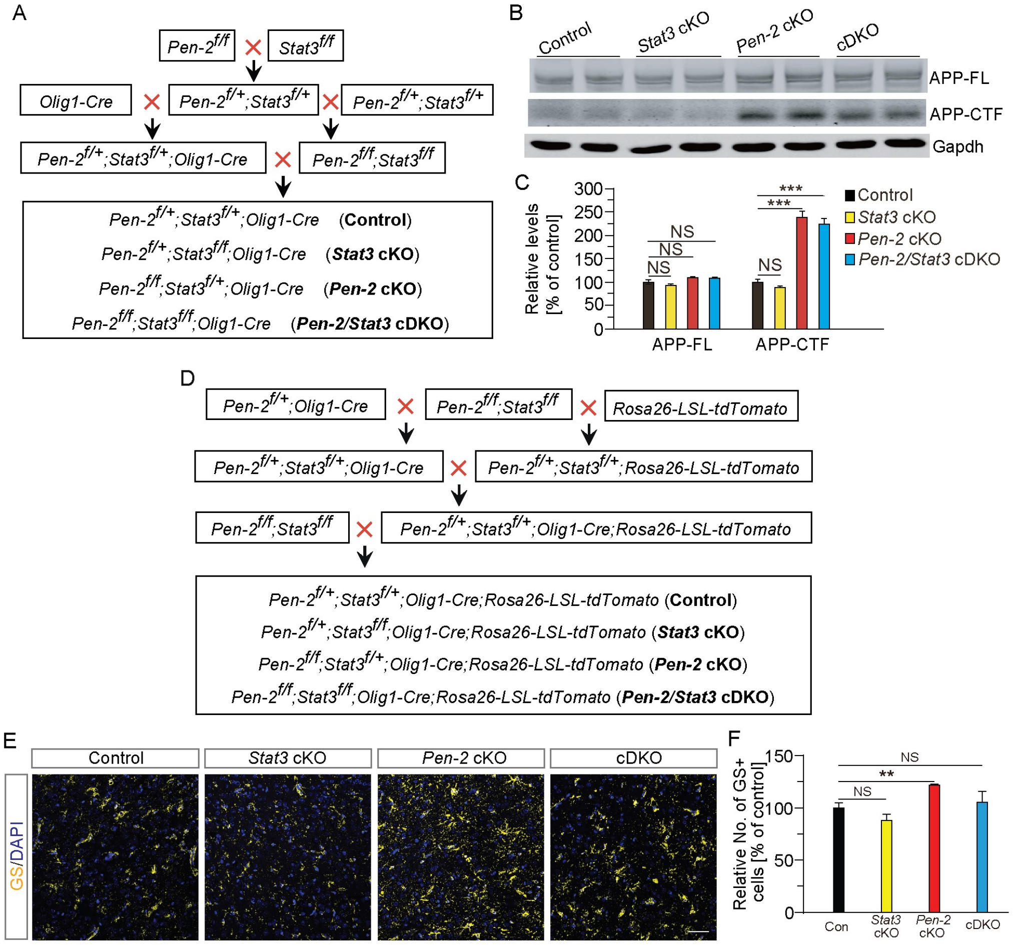

Fig. S2: Generation of OL lineage-specific *Pen-2/Stat3* cDKO mice.

A. Breeding strategy for OL lineage-specific *Pen-2/Stat3* cDKO mice. *Pen-2$^{f/f}$;Stat3$^{f/f}$* mice were crossed to *Pen-2$^{f/+}$;Stat3$^{f/+}$;Olig1-Cre* mice. The genotypes for each group are as follows: *Pen-2$^{f/f}$;Stat3$^{f/f}$* and *Pen-2$^{f/+}$;Stat3$^{f/+}$;Olig1-Cre* (Control), *Pen-2$^{f/+}$;Stat3$^{f/f}$;Olig1-Cre* (*Stat3* cKO), *Pen-2$^{f/f}$;Stat3$^{f/+}$;Olig1-Cre* (*Pen-2* cKO), and *Pen-2$^{f/f}$;Stat3$^{f/f}$;Olig1-Cre* (*Pen-2/Stat3* cDKO). **B-C.** Western blotting analysis of APP-FL and APP-CTF. Cortical samples from mice at P14 were used (B). Levels of APP-CTF were significantly elevated in *Pen-2* cKO and *Pen-2/Stat3* cDKO mice compared with littermate controls (C). There was no significant change in APP-FL levels in *Pen-2/Stat3* cDKO mice compared with controls (C) (n = 3-4 mice per group; ***, $P < 0.005$; NS, not significant). **D.** Breeding strategy for generating mice expressing tdTomato. *Pen-2$^{f/f}$;Stat3$^{f/f}$* mice were crossed to *Rosa26-LSL-tdTomato* mice. All four groups of mice, including Control, *Pen-2* cKO, *Stat3* cKO and *Pen-2/Stat3* cDKO, expressed tdTomato in a Cre-dependent manner. **E.** Representative fluorescence IHC images for GS. The scale bar is 50 μm. **F.** Quantification result. There was significantly decreased number of GS+ cells in *Pen-2/Stat3* cDKO mice compared with *Pen-2* cKO mice at P30 (n = 4-6 Mice per group; **, $P < 0.01$).

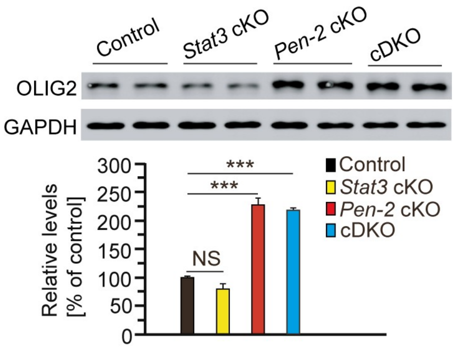

Fig. S3: Elevated levels of OLIG2 in *Pen-2/Stat3* cDKO mice.

Western blotting analysis of OLIG2. Cortical samples from four groups of mice at P14 were used. There was no significant difference in OLIG2 levels between *Pen-2* cKO and *Pen-2/Stat3* cDKO mice (n = 3-4 mice per group; ***, $P < 0.005$; NS, not significant).

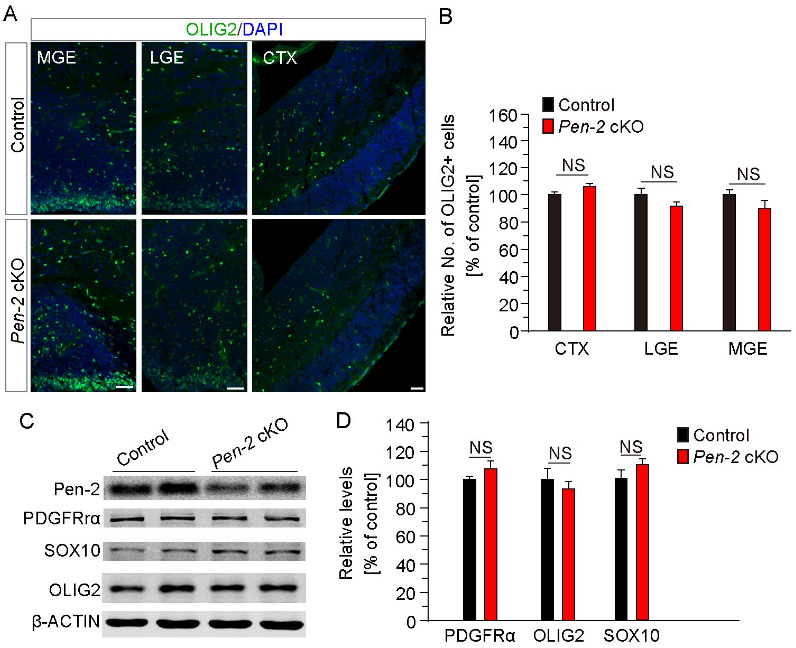

Fig. S4: No significant change in the OPC population in *Pen-2* cKO mice at E16.5.

A. Representative fluorescence IHC images for OLIG2. Brain sections were prepared from embryos at E16.5. Images are shown for MGE (median ganglion eminence), LGE (lateral ganglion eminence) and the cortex. The scale bar is 50 μm. **B.** Relative number of OLIG2+ cells [% of the control]. Cells were counted in the above brain sub-regions. There was no significant difference between control and *Pen-2* cKO mice at E16.5 (Control: n = 6; *Pen-2* cKO: n = 5; NS, not significant). **C-D.** Western blotting analysis of PDGFRα, SOX10 and OLIG2. Cortical samples from control and *Pen-2* cKO mice at E17.5 were used (C). There were no significant differences in levels of PDGFRα, SOX10 and OLIG2 between control and *Pen-2* cKO mice (D) (Control: n = 3; *Pen-2* cKO: n = 4; NS, not significant). β-ACTIN was used as the loading control.

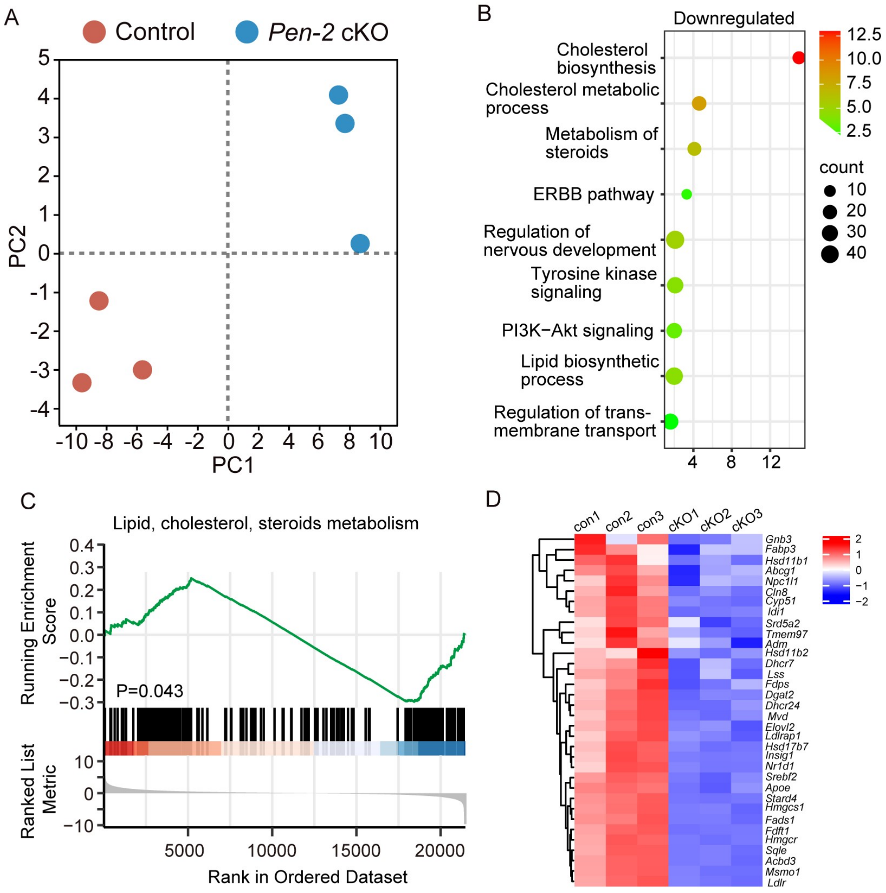

Fig. S5: Transcriptomic analysis of OPCs derived from *Pen-2* cKO mice.

A. Principal Component Analysis (PCA) of expression profiles of DEGs. PCA segregated the control and *Pen-2* cKO groups into distinct clusters, indicative of crucial expression changes associated with their respective genotypes. **B.** Gene ontology (GO) analysis for control and *Pen-2* cKO OPCs showing enriched biological processes for downregulated genes (*P*< 0.05, -$\log_2$ (fold change) > 0.5). **C.** Gene set enrichment analysis (GSEA) revealed a notable decline in the enrichment of cholesterol, steroids and lipid metabolism-related genes in *Pen-2* cKO OPCs. **D.** Heatmap of DEGs related to cholesterol and steroids metabolism.

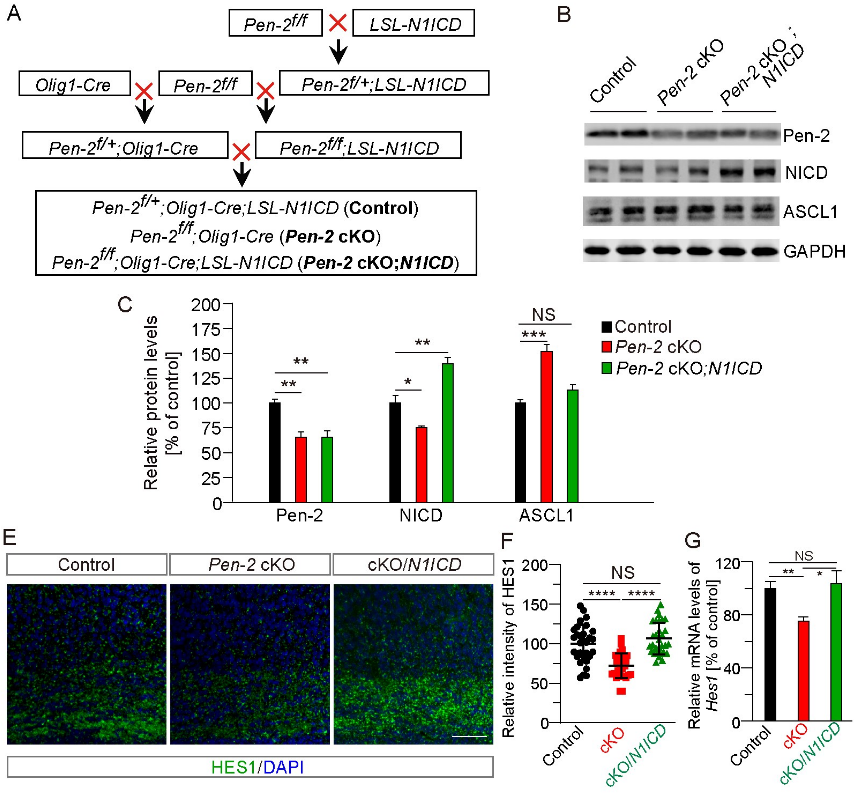

Fig. S6: Pen-2 regulates the expression of *Ascl1* via HES1.

A. Breeding strategy for generating mice expressing Notch1 ICD. The following groups of mice were used: Control (*Pen-2*$^{f/+}$*;Olig1-Cre;LSL-N1ICD*), *Pen-2* cKO (*Pen-2*$^{f/f}$*;Olig1-Cre*) and *Pen-2* cKO;*N1ICD* (*Pen-2*$^{f/f}$*;Olig1-Cre;LSL-N1ICD*). **B-C.** Western blotting analysis of PEN-2, NICD and ASCL1. Cortical samples from three groups of mice: control, *Pen-2* cKO and *Pen-2* cKO;*N1ICD* at P0 were used (B). Protein levels of PEN-2 were significantly decreased in *Pen-2* cKO and *Pen-2* cKO;*N1ICD* mice compared with controls (C). Protein levels of NICD were significantly elevated in *Pen-2* cKO;*N1ICD* mice compared with *Pen-2* cKOs (C). Protein levels of ASCL1 were significantly decreased in *Pen-2* cKO;*N1ICD* mice compared with *Pen-2* cKOs (C) (n = 4 mice per group; *, *P*< 0.05; **, *P*< 0.01; ***, *P*< 0.001; NS, not significant). **E.** Representative fluorescence IHC images for HES1 in the cortex. Brain sections were prepared from mice at P0. **F.** Relative fluorescence intensity of HES1. HES1 expression was significantly increased in *Pen-2* cKO;*N1ICD* mice compared with *Pen-2* cKOs (n = 3 mice per group, 10 HES1+ cells were randomly chosen for the measurement for each mouse; ****, P < 0.0001). **G.** qRT-PCR analysis showing increased mRNA levels of *Hes1* in *Pen-2* cKO;*N1ICD* mice compared with *Pen-2* cKOs at P0 (n = 4 mice per group; **, P < 0.01; *, P < 0.05).

Supplemental Methods

Animals

Cre-dependent breeding strategies (Ho et al., 2006; Hou et al., 2021) were used to generate *Pen-2* cKO (*Pen-2*$^{f/f}$*;Olig1-Cre*) and *Pen-2*$^{f/f}$;*Olig1-Cre*;*LSL-tdTomato* mice. To generate *Ncstn* cKO (*Ncstn*$^{f/f}$;*Olig1-Cre*) mice, *Olig1-Cre* mutants were bred with *Ncstn*$^{f/f}$ mice (Hou et al., 2016) to obtain *Ncstn*$^{f/+}$;*Olig1-Cre* mice. The latter were then crossed to *Ncstn*$^{f/f}$ to get *Ncstn* cKO mice. Since no significant differences in cell numbers for different markers were observed between *Pen-2*$^{f/f}$ and *Pen-2*$^{f/+}$*;Olig1-Cre* mice or between *Ncstn*$^{f/f}$ and *Ncstn*$^{f/+}$;*Olig1-Cre* mice, these groups were pooled to serve as controls for *Pen-2* cKO or *Ncstn* cKO mice, respectively. *Stat3*$^{f/f}$ mice were previously described (Moh et al., 2007). To generate *Pen-2/Stat3* cDKO (*Pen-2*$^{f/f}$*;Stat3*$^{f/f}$*;Olig1-Cre*) mice, *Stat3*$^{f/f}$ mice were crossed with *Pen-2*$^{f/+}$;*Olig1-Cre* mice to obtain *Pen-2*$^{f/+}$*;Stat3*$^{f/+}$*;Olig1-Cre* and *Pen-2*$^{f/+}$*;Stat3*$^{f/+}$ mice, which were subsequently intercrossed to generate *Pen-2/Stat3* cDKO mice. To generate *Pen-2*$^{f/f}$;*Olig1-Cre;LSL-N1ICD* mice, *LSL-N1ICD* mice (Cheng et al., 2019; Xia et al., 2022) were bred with *Pen-2*$^{f/f}$ to obtain *Pen-2*$^{f/+}$*;LSL-N1ICD* mice. These mice were then crossed with *Pen-2*$^{f/f}$ mice to generate *Pen-2*$^{f/f}$*;LSL-N1ICD* mice. The *Pen-2*$^{f/f}$*;LSL-N1ICD* were subsequently crossed with *Pen-2*$^{f/+}$;*Olig1-Cre* mice to produce *Pen-2*$^{f/+}$;*Olig1-Cre*;LSL-*N1ICD* (control) and *Pen-2*$^{f/f}$;*Olig1-Cre*;LSL-*N1ICD* (*Pen2 cKO;N1ICD*) mice. Genotyping was performed using tail DNAs and PCR. To detect the floxed *Pen-2* allele, the following primers were used: GACCCGTAGAAGAGCAGTCAGT (forward) and ATAAAGAATAG GCTGGGTGGTG (reverse). To detect the floxed *Ncstn* allele, the following primers were used: AGCTCTTCACCAGGTAAGAAC (forward) and TTGGACAGTCCTTCCCTGAAG (reverse). To detect the floxed *Stat3* allele, the following primers were used: TTGACCTGTGCTCCTACAAAAA (forward) and CCCTAGATTAGGCCAGCACA (reverse). The genetic background of the mice used in this study was C57BL/6. Both male and female mice were included in the experiments. Mice were group-housed (four to five mice per cage) throughout the study and provided with free access to food and water. They were maintained in a specific pathogen-free (SPF) facility at the Model Animal Research Center (MARC), Nanjing University. The animal room was equipped with an automated light cycle (12-hour light/12-hour dark) and maintained at constant humidity and temperature (25 ± 1°C).

Nissl staining

Mice were anesthetized and perfused with cold phosphate-buffered saline (PBS). The dissected brains were fixed in 4% paraformaldehyde (PFA) for 24 hours at 4°C and subsequently dehydrated using a graded ethanol series. After embedding in paraffin, each block was sectioned sagittally at a thickness of 10 μm using a microtome (Leica Microsystems, Bannockburn, IL, United States). Sections were deparaffinized with xylene and rehydrated using a graded ethanol series. Sections were stained with 0.1% cresyl violet for 1 minute and then washed with distilled water for 1 minute. Dried sections were sealed with neutral resin (Sinopharm Chemical Reagent Co. Ltd., Shanghai). Images were captured using a BX53 microscope (Olympus).

Immunohistochemistry (IHC)

Brain sections were deparaffinized with xylene, rehydrated through a graded ethanol series, and boiled in 0.01 mol/L sodium citrate buffer (pH = 6.0) for 25 minutes. After cooling to room temperature, sections were blocked using hydrogen peroxide (30% H_2O_2 diluted in methanol at a 10:1 ratio) for 30 minutes, followed by incubation with 5% bovine serum albumin (BSA) in PBS at room temperature for 30 minutes. Sections were then immunostained with primary antibodies overnight at 4°C. Antibody information is provided in Table S1. The next day, sections were incubated with biotin-labeled secondary antibodies (1:500 in PBS) at room temperature for 1 hour, treated with the avidin-biotin peroxidase complex (ABC kit, Vector Laboratories PK-6100), and developed with DAB (Vector Laboratories SK-4100). Sections were dehydrated with a graded ethanol series and sealed with neutral resin. For fluorescence immunostaining, sections were incubated with secondary antibodies conjugated to Alexa Fluor488 (1:500; Jackson ImmunoResearch or Abcam), Alexa Fluor594 (1:500; Thermo Fisher Scientific), Alexa Fluor633 (1:500; Thermo Fisher Scientific), Alexa Fluor649 (1:500; Bioworld technology), Alexa Fluor405 (1:500; Bioworld Technology), or Cy5 (1:500; Bioworld Technology). Nuclei were counterstained with DAPI (1 μg/mL; catalog #D9542, Sigma-Aldrich). Images were captured and analyzed using a fluorescence microscopy system (BX53, Olympus) or a ZEISS LSM-880 confocal laser-scanning microscope.

Cell counting

We used a method previously described by us (Hou et al., 2021). Briefly, three brain sections per mouse were used for IHC, and three to six mice were included for each genotype. Images for IHC experiments were captured using the 20× objective lens of a BX53 microscope (Olympus). ImageJ was used to count the total number of cells in each image covering an area of 438.6 μm × 330.2 μm, and the cell counts were then averaged across images from three brain sections.

To compare cell type-specific differences (e.g., PDGFRα+, OLIG2+, GS+, IBA1+, PDGFRβ+ or CD31+ cells) between control and cKO groups, the relative cell number [% of control] was calculated to illustrate the percentage change (increase) in the cKO group. However, for GFAP+ cells, the baseline count in control mice was very low, while cKO mice exhibited a substantial increase. Therefore, the absolute average number of GFAP+ cells was presented instead.

BrdU pulse-labeling

To label proliferating OPCs, BrdU (B5002, Sigma-Aldrich; 100 mg/kg) was intraperitoneally injected into mice at P0. Brains were collected 30 minutes after injection. Paraffin-embedded brain sections were then prepared. Three brain sections spaced 400 μm apart were stained with an anti-BrdU antibody.

Western blotting

After mice were anesthetized, cortical samples were freshly collected and were immediately placed into liquid nitrogen. Samples were stored at −80°C until use. Cortical samples were homogenized in cold radioimmunoprecipitation assay (RIPA) lysis buffer containing protease inhibitors and phosphatase inhibitors (Thermo Fisher Scientific) and then centrifugated at 12, 000rpm for 15 minutes at 4°C. The RIPA-soluble supernatants were used as total protein lysates. Normalized volumes of protein extracts (40 μg total protein) were loaded onto an 8%-15% SDS-PAGE gel (Invitrogen), separated by electrophoresis

at 80V for 2 hours, and then transferred onto a nitrocellulose membrane (Pall Corporation, 66485) at 25 volts for 2.5 hours. After blocking in 5% (w/v) non-fat milk solution (Sangon Biotech, A600669) for 1 hour at room temperature, the membranes were incubated with primary antibodies overnight at 4°C. After washing with TBS three times, the membranes were incubated with corresponding Li-Cor IRDye infrared dye-coupled secondary antibodies (goat anti-rabbit IRdye680, goat anti-mouse IRdye800, and goat anti-mouse IRdye680). The membranes were scanned, and data were analyzed using the Odyssey Infrared Imaging System (Li-Cor).

Quantitative real-time PCR

Total RNA was extracted from fresh mouse cortices using the TRIzol reagent (Invitrogen, USA) according to the manufacturer's instructions. The purity and concentration of RNA were determined using a NanoDrop ND-1000 spectrophotometer. Isolated RNA was stored at −80°C until use. Equal amounts (1 μg) of total RNA were reverse transcribed to cDNA using the PrimeScript RT reagent Kit (Takara, RR047A) according to the manufacturer’s instructions. Quantitative real-time PCR (qRT-PCR) was performed using 2× RealStar Green Fast Mixture with Rox (Genstar, A303-05) on an Applied Biosystems Prism StepOne Plus system (Thermo Fisher Scientific). PCR reactions were performed independently three times, with each sample loaded in duplicates. Quantification was performed using the comparative cycle threshold (Ct) method, with Gapdh as the internal control. Primer information was provided in Table S2.

Constructs of plasmids and lentivirus production

The construction of the pCDNA5-HA-HES1 plasmid was described previously (Hou et al., 2021). The mouse *Ascl1* promoter region, spanning from -1998 to +504 bp relative to the transcriptional start site (TSS), was amplified by PCR from mouse genomic DNA and cloned into the pGL3-luciferase vector (Promega). To knockdown *Ascl1* in OPCs, HEK293T cells were transfected with virus packaging plasmids along with either the pLKO.1 empty vector (control) or specific constructs encoding *Ascl1*-shRNA1 (CCGGAACTGATGCGCTGCAAA) or *Ascl1*-shRNA2 (CTCC AACGACTTGAACTCTAT) using Lipofectamine 2000 transfection reagent (Thermo Fisher Scientific). After 48 hours of incubation, polybrene was added to the lentiviral supernatant at a concentration of 10 μg/mL to enhance viral transduction efficiency. The supernatant was then filtered through a 0.45 μm membrane to remove cell debris, and the lentiviral particles were concentrated by ultracentrifugation at 100,000× g.

Primary OPC culture and lentivirus infection

OPCs were isolated using a previously described method (Hou et al., 2021). Briefly, OPCs were prepared from the cerebral cortices of P8 pups. After removing the meninges under a dissection microscope, cortices were dissected and dissociated by mechanical trituration. The cortical tissues were enzymatically digested with 0.25% trypsin and 10 μg/mL DNase at 37°C for 6 minutes. The digestion was terminated by adding an equal volume of DMEM supplemented with 10% FBS. The cells were then washed thoroughly with ice-cold DMEM and filtered through a 70 μm nylon cell strainer to obtain a single-cell suspension. The cell suspension was plated onto poly-D-lysine-coated T-flasks and maintained in neurosphere medium (DMEM/F12 containing 2 mM L-glutamine, 1×B27, 1×N2, 5 μM HEPES, 0.01% heparin, 100 μg/mL penicillin, 0.1 mg/mL streptomycin, 20 ng/mL EGF, 20 ng/mL FGFb and 20 ng/mL PDGF-AA) at 37°C in a

humidified incubator with 5% CO_2 for suspension culture. The culture medium was replenished every 4 days to maintain optimal growth conditions. After 12 days, OPCs were infected with lentivirus expressing GFP, *Ascl1*-shRNA1-GFP or *Ascl1*-shRNA2-GFP. 4 days post-infection, OPCs were harvested for Western blotting analysis. For OPC self-renewal assay, dividing OPCs were labeled with 10 μM BrdU for 24 hours and plated onto poly-D-lysine-coated coverslips for immunostaining.

RNA-seq analyses

Total RNA was extracted from cultured OPCs using TRIzol reagent according to the manufacturer's instructions. Genomic DNA was then meticulously removed using DNase I (Takara). The integrity and purity of the resulting total RNA were assessed using the 2100 Bioanalyzer (Agilent Technologies, Santa Clara, CA, USA), and its concentration was determined using the ND-2000 spectrophotometer (NanoDrop Thermo Scientific, Wilmington, DE, USA). Transcriptome libraries were then prepared using the Illumina TruSeq^{TM} RNA Sample Preparation Kit (San Diego, CA). Poly(A) mRNA was first purified from total RNA using oligo-dT-conjugated magnetic beads, followed by fragmentation with a fragmentation buffer. These short fragments served as templates for synthesizing double-stranded cDNA using the SuperScript Double-Stranded cDNA Synthesis Kit (Invitrogen, CA) and random hexamer primers. The synthesized cDNA underwent end-repair, phosphorylation, and the addition of an 'A' base following Illumina's library construction protocol. The libraries were then size-selected to isolate cDNA fragments (200-300 bp) on 2% Low Range Ultra Agarose gels. These fragments were then PCR-amplified for 15 cycles using Phusion DNA polymerase (New England Biolabs, Boston, MA). After quantification with TBS380, two RNA-seq libraries were sequenced in a single lane on the Illumina HiSeqX Ten/NovaSeq 6000 sequencer (Illumina, San Diego, CA), generating 2×150 bp paired-end reads. Each sample yielded over 55 million reads, which were aligned to the mouse genome database GRCm38.p4 (mm10). Only uniquely and accurately mapped read pairs were retained for downstream analyses. Gene expression was quantified using Transcripts Per Million (TPM) values calculated by StringTie (version 1.3.0), based on the number of fragments per gene after HISAT2 alignment. Differential expression analysis was performed using the EdgeR, with adjusted P-values calculated using the Benjamini-Hochberg method to control the false discovery rate. Gene with $P < 0.05$ and $|\log2 (\text{foldchange})| > 0.5$ were considered significantly differentially expressed. Hierarchical clustering analysis was performed to explore the expression patterns of differentially expressed genes (DEGs). Gene Ontology (GO) enrichment analysis was conducted using R based on DAVID v2024q1 (https://david.ncifcrf.gov/home.jsp). Finally, Gene Set Enrichment Analysis (GSEA) was performed using the online platform (https://www.bioinformatics.com.cn) (last accessed on 20 February 2024), a comprehensive resource for data analysis and visualization.

Luciferase reporter assay

The luciferase assay was performed as recently described (Hou et al., 2021). Briefly, the HA-HES1 vector was co-transfected with the *Ascl1*-driven luciferase reporter vector (*Ascl1*-Luc) and the pRL-SV40-Renilla normalization vector into HEK293T cells. After 48 hours of incubation in the incubator, the luciferase assay was conducted using the Dual-Luciferase Reporter Assay System (Promega) according to the manufacturer’s protocol. The measured Firefly luciferase activity was normalized to that of Renilla

luciferase to account for variations in transfection efficiency and cell viability. Experiments were performed in duplicate and repeated three times. Primer information was provided in Table S2.

Supplemental Tables

Table S1. Information for antibodies used in this study.

Antibodies	Source	Identifier
Rabbit anti-GFAP	Abcam	Cat# ab7260, RRID: AB_305808
Rabbit anti-PDGFRα	Cell Signaling Technology	Cat# 3174, RRID: AB_2162345
Mouse anti-OLIG2	Millipore	Cat# MABN50, RRID: AB_10807410
Mouse anti-STAT3	Cell Signaling Technology	Cat# 9139, RRID: AB_331757
Rabbit anti-NCSTN	Invitrogen	Cat# 34-9200, RRID: AB_138816
Rabbit anti-NeuN	Millipore	Cat# ABN78, RRID: AB_10807945
Rabbit anti-APP	Sigma-Aldrich	Cat# A8717, RRID: AB_258409
Rabbit anti Pen-2	Abclonal	Cat# A15172, RRID: AB_2762062
Rabbit anti-SOX10	Abcam	Cat# ab155279, RRID: AB_2650603
Rat anti-BrdU	Abcam	Cat# ab6326, RRID: AB_305426
Rabbit anti-ASCL1	ZenBio	Cat# 382207, RRID: Not available
Rabbit anti-HES1	Cell Signaling Technology	Cat# 11988, RRID: AB_2728766
Rabbit anti-HES1	Abcam	Cat# ab71559, RRID: AB_1209570
Mouse anti-GAPDH	Abcam	Cat# ab8245, RRID: AB_2107448
Mouse anti-β-ACTIN	Sigma-Aldrich	Cat# A1978, RRID: AB_476692
Rabbit anti-IBA1	Wako	Cat# 019-19741, RRID: AB_839504
Goat anti-PDGFRβ	R and D Systems	Cat# AF1042, RRID: AB_ 2162633
Rat anti-CD31	BD Biosciences	Cat# 550274, RRID: AB_ 393571
Rabbit anti-GS	Abcam	Cat# ab73593, RRID: AB_2247588
Alexa Fluor® 488 AffiniPure Goat Anti-Rabbit IgG (H+L)	Jackson Immuno Research Laboratories	Cat# 111-545-003, RRID: AB_2338046
Alexa Fluor® 488 AffiniPure Donkey Anti-Mouse IgG (H+L)	Jackson Immuno Research Laboratories	Cat# 715-545-150, RRID: AB_2340846
Alexa Fluor™633 Goat Anti-Rat IgG (H+L)	Thermo Fisher Scientific	Cat# A-21094, RRID: AB_2535749
Alexa Fluor™ 594 Donkey Anti-Mouse IgG (H+L)	Thermo Fisher Scientific	Cat# A-21203, RRID: AB_2535789
Donkey Anti-Rat IgG H&L(Alexa Fluor® 488) preabsorbed	Abcam	Cat# ab150153, RRID: AB_2737355
Dylight 649-conjugated AffiniPure Goat Anti- Rabbit IgG (H+L)	Bioworld technology	Cat# BS10034, RRID: Not available
Dylight 405-conjugated AffiniPure Goat Anti- Rat IgG (H+L)	Bioworld technology	Cat# BS10014, RRID: Not available
Goat Polyclonal Secondary Antibody to Mouse IgG (H&L) Cy5	Bioworld technology	Cat# BS22101, RRID: Not available

labeled		
DAPI	Sigma-Aldrich	Cat# D9542
IRDye® 800CW Goat Anti-Mouse IgG (H+L) antibody	LI-COR Biosciences	Cat# 926-32210, RRID: AB_621842
IRDye 680RD Donkey Anti-Mouse IgG (H+L) antibody	LI-COR Biosciences	Cat# 926-68072, RRID: AB_10953628
IRDye 680RD Goat Anti-Rabbit IgG (H+L) antibody	LI-COR Biosciences	Cat# 926-68071, RRID: AB_10956166
Biotin-SP-AffiniPure Goat Anti-Mouse IgG (H+L) antibody	Jackson Immuno Research Laboratories	Cat# 115-065-003, RRID: AB_2338557
Biotin-SP-AffiniPure Goat Anti-Rabbit IgG (H+L) antibody	Jackson Immuno Research Laboratories	Cat# 111-065-003, RRID: AB_2337959

Table S2. Information for primers used in this study.

Primers for qRT-PCR		
Gene	Species	Sequence 5'-3'
Pen-2	Mouse	Forward: TGGATTTGCGTTCCTGCCTTTTCT
		Reverse: ATGAAGTTGTTAGGGAGTGCC
Ascl1	Mouse	Forward: TCCTACGACCCTCTTAGCCC
		Reverse: CATTTGACGTCGTTGGCGAG
Id4	Mouse	Forward: CACCCTGCTTTGCTGAGAC
		Reverse: CAGAGAATGCTGTCACCCTG
Bmp4	Mouse	Forward: TCTCCGTCCCTGATGGGATT
		Reverse: AAACGACCATCAGCATTCGGTTA
Gapdh	Mouse	Forward: AATGTGTCCGTCGTGGATCT
		Reverse: CCCTGTTGCTGTAGCCGTAT
Primers for promoter		
Promoter	Region	Sequence 5'-3'
m*Ascl1*-promoter	-1,998 bp~+504 bp	Forward: CTCTGCTTCTGTGGGGAGTG
		Reverse: GGGGCTGCGAAGCACGATCA
m*Ascl1*-P1	-1,973 bp~-1,629 bp	Forward: AGCTGTGGGGCAGGTAAGGA
		Reverse: CATTGGTCACCACTTCTGGC
m*Ascl1*-P2	-1,584 bp~ -1,286 bp	Forward: AGAGGCCAGCTTAGGCTATG
		Reverse: CTCTGTCCGAACCCAGTCCA
m*Ascl1*-P3	-1,220 bp~ -944 bp	Forward: AGGCACCTCCCCTTCTCTCG
		Reverse: GAAAGATGCTTGCTAGACGC
m*Ascl1*-P4	-741 bp~ -431 bp	Forward: TCCTTGAAGCTGAATGGAAC
		Reverse: TTCAGGGAAGGGTTTAGGCA
m*Ascl1*-P5	-393 bp~ -25 bp	Forward: TTCCCTCCAGACTTTCCACC
		Reverse: TTCAATGGGACACCCAGCCC
m*Ascl1*-P6	+134 bp~ +500 bp	Forward: TCAAGCCCAGGCTGGAGCAA
		Reverse: CTGCGAAGCACGATCAAAGG

REFERENCES

Cheng, S., Liu, T., Hu, Y., Xia, Y., Hou, J., Huang, C., Zou, X., Shi, Y., Zheng, Y., Lu, J., and Chen, G. (2019). Conditional inactivation of Pen-2 in the developing neocortex leads to rapid switch of apical progenitors to basal progenitors. J Neurosci *39*, 2195-2207.

Ho, A., Morishita, W., Atasoy, D., Liu, X., Tabuchi, K., Hammer, R. E., Malenka, R. C., and Sudhof, T. C. (2006). Genetic analysis of Mint/X11 proteins: essential presynaptic functions of a neuronal adaptor protein family. J Neurosci *26*, 13089-13101.

Hou, J., Bi, H., Ye, Z., Huang, W., Zou, G., Zou, X., Shi, Y., Shen, Y., Ma, Q., Kirchhoff, F., *et al.* (2021). Pen-2 negatively regulates the differentiation of oligodendrocyte precursor cells into astrocytes in the central nervous system. J Neurosci *41*, 4976-4990.

Hou, J., Cheng, S., Chen, L., Wang, Q., Shi, Y., Xu, Y., Yin, Z., and Chen, G. (2016). Astroglial activation and tau hyperphosphorylation precede to neuron loss in a neurodegenerative mouse model. CNS Neurosci Ther *22*, 244-247.

Moh, A., Iwamoto, Y., Chai, G. X., Zhang, S. S. M., Kano, A., Yang, D. D., Zhang, W., Wang, J., Jacoby, J. J., Gao, B., *et al.* (2007). Role of STAT3 in liver regeneration: survival, DNA synthesis, inflammatory reaction and liver mass recovery. Lab Invest *87*, 1018-1028.

Xia, Y., Zhang, Y., Xu, M., Zou, X., Gao, J., Ji, M., and Chen, G. (2022). Presenilin enhancer2 is crucial for the transition of apical progenitors into neurons but into not basal progenitors in the developing hippocampus. Development *149*, dev.200272.
